# Effects of amino acids on the lignocellulose degradation by *Aspergillus fumigatus* Z5: insights into performance, transcriptional, and proteomic profiles

**DOI:** 10.1186/s13068-018-1350-2

**Published:** 2019-01-04

**Authors:** Jiaxi Miao, Mengmeng Wang, Lei Ma, Tuo Li, Qiwei Huang, Dongyang Liu, Qirong Shen

**Affiliations:** 1 Jiangsu Key Lab for Organic Solid Waste Utilization, Nanjing, 210095 China; 2 Jiangsu Collaborative Innovation Center for Solid Organic Waste Resource Utilization, Nanjing, 210095 China; 3 College of Resources and Environmental Science, Nanjing Agricultural University, Nanjing, 210095 China

**Keywords:** Lignocellulose, *Aspergillus fumigatus* Z5, Secretome, Amino acids, Transcriptome, Proteome

## Abstract

**Background:**

As a ubiquitous filamentous fungal, *Aspergillus* spp. play a critical role in lignocellulose degradation, which was also defined as considerable cell factories for organic acids and industrially relevant enzymes producer. Nevertheless, the production of various extracellular enzymes can be influenced by different factors including nitrogen source, carbon source, cultivation temperature, and initial pH value. Thus, this study aims to reveal how amino acids affect the decomposition of lignocellulose by *Aspergillus fumigatus* Z5 through transcriptional and proteomics methods.

**Results:**

The activities of several lignocellulosic enzymes secreted by *A. fumigatus* Z5 adding with cysteine, methionine, and ammonium sulfate were determined with the chromatometry method. The peak of endo-glucanase (7.33 ± 0.03 U mL^−1^), exo-glucanase (10.50 ± 0.07 U mL^−1^), β-glucosidase (21.50 ± 0.22 U mL^−1^), and xylanase (76.43 ± 0.71 U mL^−1^) were all obtained in the Cys treatment. The secretomes of *A. fumigatus* Z5 under different treatments were also identified by LC–MS/MS, and 227, 256 and 159 different proteins were identified in the treatments of Cys, Met, and CK (Control, treatment with ammonium sulfate as the sole nitrogen source), respectively. Correlation analysis results of transcriptome and proteome data with fermentation profiles showed that most of the cellulose-degrading enzymes including cellulases, hemicellulases and glycoside hydrolases were highly upregulated when cysteine was added to the growth medium. In particular, the enzymes that convert cellulose into cellobiose appear to be upregulated. This study could increase knowledge of lignocellulose bioconversion pathways and fungal genetics.

**Conclusions:**

Transcriptome and proteome analyses’ results indicated that cysteine could significantly promote the secretion of lignocellulosic enzymes of an efficient lignocellulosic decomposing strain, *A. fumigatus* Z5. The possible reason for these results is that Z5 preferred to use amino acids such as cysteine to adapt to the external environment through upregulating carbon-related metabolism pathways.

**Electronic supplementary material:**

The online version of this article (10.1186/s13068-018-1350-2) contains supplementary material, which is available to authorized users.

## Background

Various saprotrophic filamentous fungi own a considerable capacity of lignocellulose-degrading efficiency, which is considered as the most abundant natural materials, and it is the most abundant resource present in a variety of plants that humans can easily access and use. The growing focus on depleting fossil fuels requires a shift from nonrenewable carbon sources to renewable biological resources such as lignocellulose. Regardless of the cause, lignocellulosic materials consist of three main polymers: cellulose (a glucose homopolymer), hemicellulose, (heteropolymers of pentoses and hexoses), and lignin (phenyls, amorphous polymers) [1]. Approximately 180 billion tons of cellulose are produced annually by plants, making this polysaccharide a substantial organic carbon pool on earth [2]. It is one of the most widely distributed and most abundant substances on earth and one of the cheapest renewable resources. Plant cellulose is mainly degraded by various microorganisms into organic carbon sources and then transformed into the most substantial material flows in the biosphere. Therefore, the importance of cellulose as a renewable energy source has become the subject of research and commerce. Nevertheless, the critical step in the use of cellulose is its hydrolysis into monomeric sugars and its eventual conversion to valuable chemicals and energy [3].

Lignocellulolytic enzymes are a series of enzymes related to lignocellulose degradation, including pectinases, cellulases, hemicellulases, manganese peroxidase (MnP), lignin peroxidase (LiP), and laccase (Lac), [4]. As the major components of lignocellulolytic enzymes, cellulase consists of at least three types of enzymes: endo-glucanases (EC 3.2.1.4) which act randomly on insoluble and soluble cellulose chains; exo-glucanases (cellobiohydrolases EC 3.2.1.91), which respond to liberate cellobiose from the reducing and nonreducing ends of cellulose chains; and β-glucosidases (EC 3.2.1.21), which liberate glucose from cellobiose [5]. Each component contains multiple isoenzymes, such as the *Trichoderma reesei* cellulase system, including at least five endonucleases (EGI–EGV), two exonucleases (CBHI, CBHII), and two β-glucosidases (BGI, BGII). Enzymes degrading the hemicelluloses (called hemicellulases) are well characterized, and are classified according to their substrate specificities, such as xylanase, lichenase, and laminarinase. Pectinase is an enzyme that can break down pectin. The degradation of lignocellulose requires the synergistic action of all these enzymes mentioned above, especially cellulases and hemicellulases.

Most of the hydrolytic enzymes are secreted by various microbes, including bacteria, actinomycetes, and filamentous fungi, which have been screened from various habitats [1, 6]. Among the different methods of utilizing lignocellulose, microbial degradation technology has attracted a large amount of attention worldwide because of its advantages of having low cost, employing mild reaction conditions, and lack of pollution to the environment [7]. Due to high extracellular enzymatic activity and a relatively large number of enzymatic species, fungi have a considerable capacity to degrade cellulose. Meanwhile, the fungi can contribute significantly to recycling lignocellulosic biomass due to their capacities of secreting a large number of lignocellulolytic enzymes [8]. Therefore, filamentous fungi, including *Trichoderma*, *Aspergillus*, *Penicillium*, *Acremonium*, *Myrothecium*, *Neurospora*, and *Chaetomium*, have been extensively applied in the cellulose industry. *A. fumigatus* Z5 can efficiently decompose various agricultural enzymes with the help of cellobiohydrolases which belong to the glycoside hydrolase (GH) families and carbohydrate-active enzymes (CAZy) including GH1, GH3, GH5, GH9, GH12, GH44, GH45, and lytic polysaccharide monooxygenases (LPMO) [9, 10]. In addition, *A. fumigatus* Z5 genomes encode many other CAZy such as polysaccharide lyases (PLs) and carbohydrate esterases (CEs), for the degradation hemicelluloses and pectin [10].

Many studies on the microbial degradation of lignocellulose have mainly focused on microbial resources, enzyme properties and synthetic regulation, and enzyme genetic engineering [11, 12]. However, selection of the specific nutritional factors that influence the biodegradation ability of lignocellulosic fungi and its concrete mechanisms is still rarely reported at present. Nitrogen sources are indispensable during the secretion process of various extracellular enzymes by *A. fumigatus* Z5, especially for specific kinds such as amino acids and peptides. As one of the important nutritional factors, amino acids, and their analogs are known to stimulate the enzyme production of various fungi, such as α-amylase and xylanases [13, 14]; unfortunately, this biological mechanism is not yet clear. Here, amino acids were added into culture medium containing rice straw powder, and the effect of amino acids on the cellulose production of *A. fumigatus* Z5 was explored to reveal the intrinsic mechanism through the combination of transcriptome and proteome analysis methods, which can reveal the specific lifestyle of each fungal species and the strategy that each species utilizes for lignocellulose conversion [15–17]. Transcriptomics can help reveal a synergistic response of a fungal strain to the external environment and nutritional changes, and proteomics is a useful tool to discover profile and identify various proteins in response to special environment.

The objective of this study is to reveal how amino acids (cysteine and methionine) affect lignocellulose biodegradation by the efficient lignocellulose-degrading strain *A. fumigatus* Z5. Moreover, the RNA-seq transcriptome profiles and the 4-plex 2D HPLC–MS/MS quantitative proteomic profile were applied to analyze the genes involved in substrate degradation and cellodextrin transport to reveal the intrinsic biological mechanism. Overall findings improve our knowledge of the biodegradation mechanisms of lignocellulosic fungi, and it is anticipated that this knowledge will have benefits for the development of biofuel production.

## Results

### Effects of various pure amino acids on the cellulase production of *A. fumigatus* Z5

**Fig. 1:**
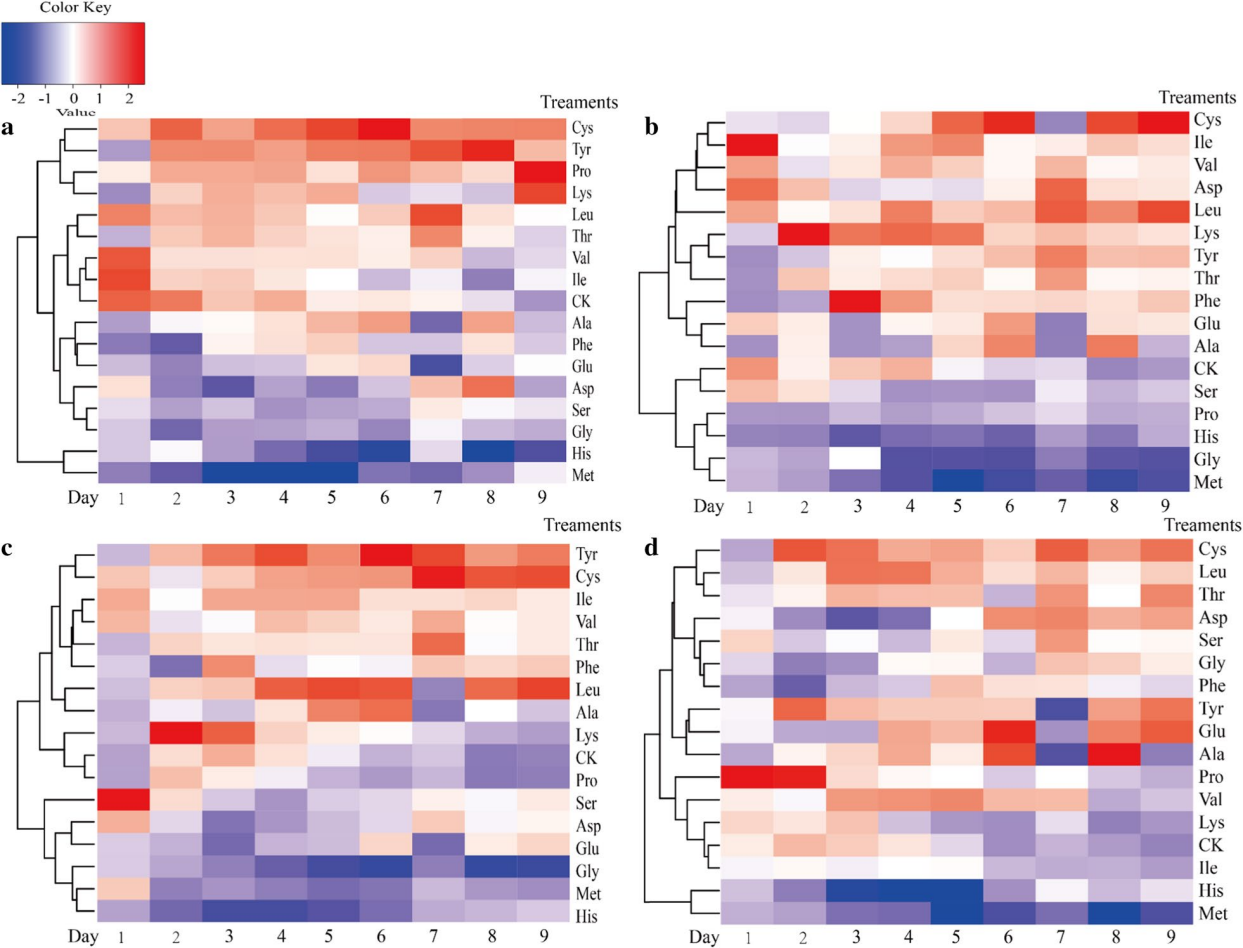
Activities of extracellular hydrolytic enzymes in the secretome of *A. fumigatus* Z5 in the presence of different amino acids. **a** Time course profiles of endo-glucanase activities of different treatments under the regulation of various amino acids; **b** time course profiles of exo-glucanase activities of different treatments under the regulation of various amino acids; **c** time course profiles of β-glucosidase activities of different treatments under the regulation of various amino acids; **d** time course profiles of xylanase activities of different treatments under the regulation of various amino acids

Various pure amino acids as indicated in the experimental procedures were used as specific nutritional factors to evaluate the biodegradation of rice straw by *A. fumigatus* Z5, and the endo-glucanases, exo-glucanases, β-glucosidases, and xylanase activities were determined during nine consecutive days after inoculation. The changes in enzyme activities were mainly consistent among different treatments. The enzyme activity stayed extremely low during the first 3 days, and the maximal values were obtained around the 6th day. Most notably, cellulase activity significantly increased after supplying cysteine, and the maximal activities for endo-glucanase, exo-glucanase, β-glucosidase, and xylanase were 7.33 ± 0.03 U mL^−1^, 10.50 ± 0.07 U mL^−1^, 21.50 ± 0.22 U mL^−1^, and 76.43 ± 0.71 U mL^−1^, respectively (Fig. 1). Unlike the Cys treatment, the addition of methionine caused a decrease in cellulase activities, and the enzyme activities of endo-glucanase, exo-glucanase, β-glucosidase, and xylanase were 3.68 ± 0.07 U mL^−1^, 1.48 ± 0.04 U mL^−1^, 3.73 ± 0.04 U mL^−1^, and 39.20 ± 0.65 U mL^−1^, respectively. For the CK treatment, the enzyme activities of endo-glucanase, exo-glucanase, β-glucosidase, and xylanase were 5.65 ± 0.01 U mL^−1^, 4.55 ± 0.01 U mL^−1^, 10.48 ± 0.21 U mL^−1^, and 51.30 ± 0.56 U mL^−1^, respectively. On the other hand, different concentrations of cysteine and methionine (0.5, 1.0, 1.5, 2.0, 2.5, and 3.0 g L^−1^) were added to evaluate the effects on the biodegradation of lignocellulose, and the results are shown in Additional file 1: Figure S2. The results indicated that the optimum concentrations for endo-glucanase and exo-glucanase activities were 1.5 g L^−1^ for Cys treatment, while the optimum concentrations for xylanase activity were 2.0 g L^−1^. Meanwhile, the optimum concentration for a negative effect on all the detected enzyme activities was 2.0 g L^−1^. Therefore, cysteine and methionine were chosen for further investigation for their effects on the biodegradation of lignocellulose by *A. fumigatus* Z5.

**Fig. 2:**
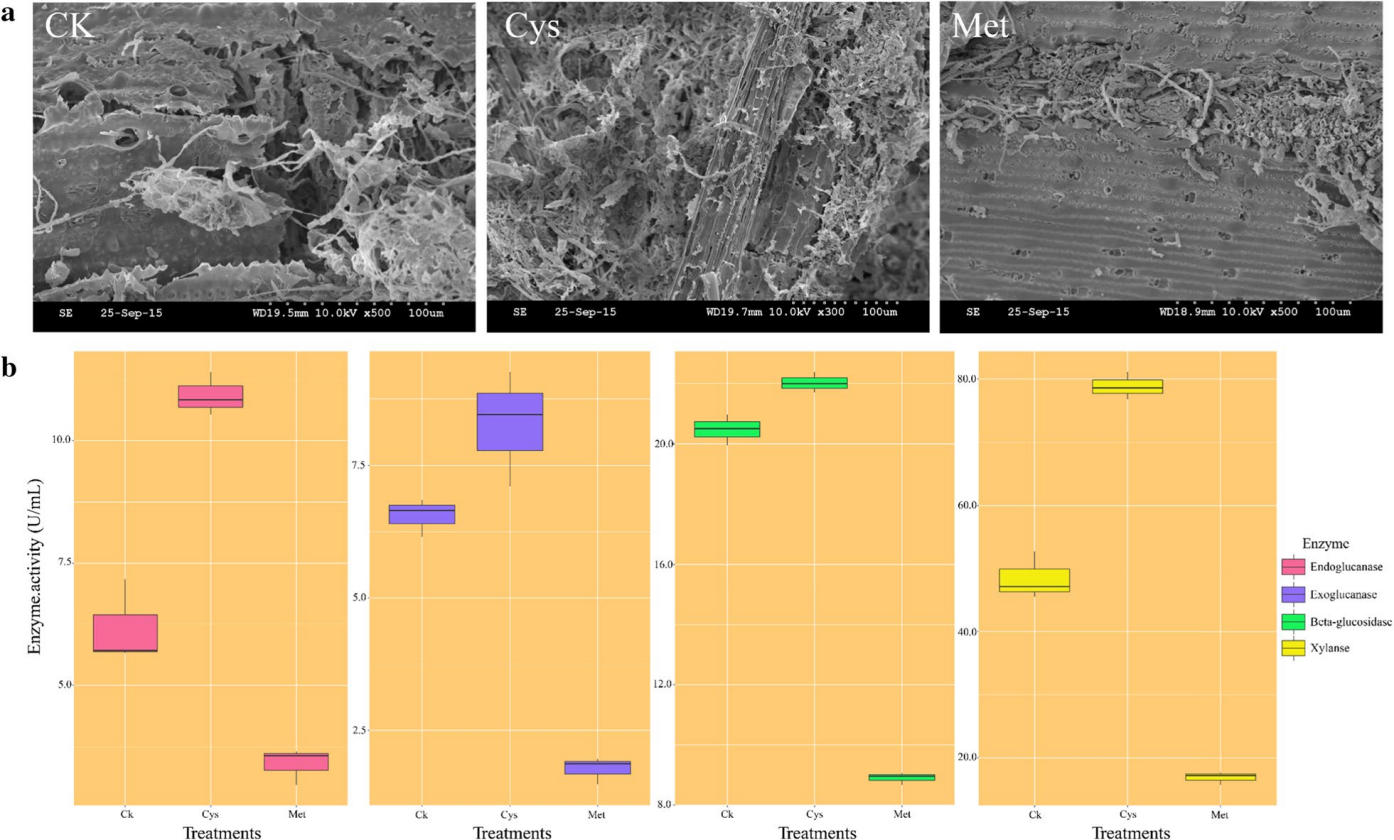
Growth and extracellular hydrolytic enzyme activities of *A. fumigatus* Z5 with the regulation of cysteine and methionine using rice straw as sole carbon source. **a** Scanning electron microscope images of the substrates after 4 days of degradation by *A. fumigatus* Z5 under solid-state fermentation with the regulation of different amino acids; **b** changes in the activities of endo-glucanase, exo-glucanase, β-glucosidase, and xylanase in different treatments

Mycelial growth was more extensive in the Cys treatment than in the CK treatment. However, mycelial growth is minimal and almost invisible in the Met treatment (Additional file 1: Figure S1). The highest biomass was obtained in the Cys treatment (281.92 mg g^−1^ dw), while the lowest biomass was obtained in the Met treatment (228.82 mg g^−1^ dw) (Additional file 1: Figure S3). Samples from different treatments were taken to compare the change of the surface through scanning electron microscopy (SEM). The xylem and cell wall ultrastructure with tracheid-bordered pits could be observed in raw materials (data not shown). Samples from different treatments were also observed, and the results are shown in Fig. 2a. Under the influence of cysteine, the decomposition effect of rice straw was better than that of CK, and the internal structure was visible with a large number of holes appearing on the surface. Moreover, fungal hyphae could directly enter the interior of the rice straw, resulting in easier decomposition. In the Met treatment, the surface of the rice straw was substantially unchanged compared to the raw materials, and only small cracks produced by Z5 acting on the straw surface could be observed. Hydrolytic enzyme activities, including endo-glucanase, exo-glucanase, β-glucosidase, and xylanase, were determined to evaluate the degree of degradation of agricultural wastes. All enzyme activities in the Cys treatment were significantly higher than that of CK, and the endo-glucanase activity, exo-glucanase activity, β-glucosidase activity, and xylanase activity were 11.39 ± 0.26 U mL^−1^, 9.26 ± 0.63 U mL^−1^, 22.38 ± 0.20 U mL^−1^, and 77.95 ± 1.70 U mL^−1^, respectively. All enzyme activities in Met were significantly lower than those in CK, including endo-glucanase (2.97 ± 0.22 U mL^−1^), exo-glucanase (1.48 ± 0.15 U mL^−1^), β-glucosidase (8.68 ± 0.11 U mL^−1^), and xylanase activity (17.91 ± 1.06 U mL^−1^) (Fig. 2b).

### Identification of the secretomes from different supernatants under solid-state fermentation

**Fig. 3:**
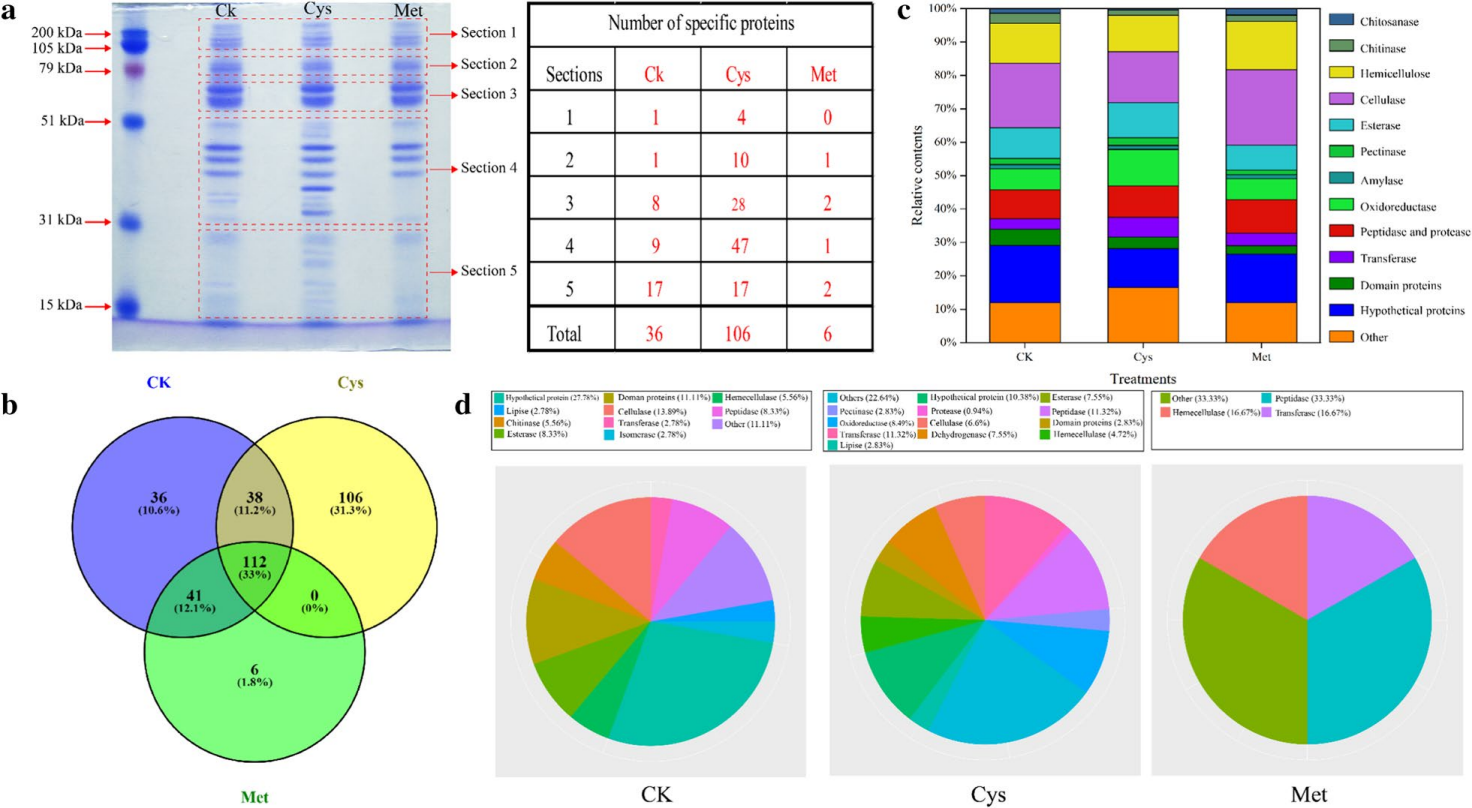
SDS-PAGE and functional analysis of the proteins secreted by *A. fumigatus* Z5 under solid-state fermentation in different treatments. **a** SDS-PAGE analysis of the secretomes from *A. fumigatus* Z5 in different treatments; **b** Venn diagrams of various proteins identified in the secretome of *A. fumigatus* Z5 from different treatments; **c** relative contents of various proteins identified in different supernatants; **d** functional classification of specific proteins in different treatments

SDS-PAGE analysis results of concentrated culture supernatants from different treatments are shown in Fig. 3a. Secretomes from *A. fumigatus* Z5 were also found to be dependent on the different amino acids, and all the bands in different treatments were separated into five sections. The number of specific proteins in different lanes was detected, among which Sect. 4 had the majority of the components from each treatment, and the number of specific proteins in this section was 9, 47, and 1, corresponding to CK, Cys, and Met, respectively. A total of 339 proteins were identified in the secretome of the different treatments through database searches (NCBInr) under SSF conditions (Fig. 3b). Figure 3b shows a Venn diagram illustrating the number of proteins identified in different treatments. A total of 227 proteins were detected for CK, 256 proteins were identified for Cys, and 159 proteins were detected for Met. Thirty-six, 106, and 6 proteins were identified as unique for control treatment, cysteine treatment, and methionine treatment, respectively. Seventy-nine proteins were partially shared among two of the three treatments, and 112 proteins were common to all treatments (Additional file 2: Dataset S1).

As depicted in Fig. 3c, the proteins were divided into cellulose-, hemicellulose-, pectin-, chitin-, lipid-, starch-, or protein-degrading enzymes and oxidoreductase, peptidase, dehydrogenase or transport proteins. The complete list of proteins secreted by Z5 is presented in Additional file 1: Table S1. Cellulase, hemicellulase, chitinase, and chitosanase formed the largest class of proteins which comprised 21%, 18%, and 17% of the identified proteins for CK, Cys, and Met, respectively. A significant portion of the identified proteins in this study was hypothetical proteins or with an unknown function, which meant that the peptide sequence matched either an ORF that had not previously been shown to be expressed or a protein with unknown function. This category also contains 12%, 9%, and 7% of the identified proteins of CK, Cys, and Met, respectively. Proteins involved in peptidolysis and proteolysis were also highly abundant, comprising 6%, 7%, and 5% of the identified proteins of CK, Cys, and Met, respectively. Moreover, the remainder of the identified proteins were functionally diverse such as oxidoreductase and transferase. Thirty-six proteins were unique to CK, and cellulase and hemicellulase comprised 12% of these identified proteins; 106 proteins were novel in Cys, cellulase, and hemicellulase comprised 20% of the identified proteins; only six proteins were individual to Met, two of which were involved in proteolysis and peptidolysis, and one of which was involved in carbohydrate metabolism. In particular, only Cys contained a significant amount of oxidoreductase compared to the other treatments (Fig. 3d).

The identified secretomes from *A. fumigatus* Z5 in different treatments are shown in Additional file 1: Table S1. A total of 129 proteins containing signal peptides were identified in the secretomes, and most of the proteins were involved in lignocellulose degradation. All of the proteins identified in the extracellular crude enzymes contained a signal peptide, and the total number of cellulases, hemicellulases, and chitinases was 66. In addition to the several enzymes mentioned above, 11 pectin lyases were identified, and some esterases, catalases, and transferases were also detected. All of the proteins with signal peptides were separated into five different sections according to molecular size, and most of the lignocellulosic enzymes were distributed in Sect. 4 with a molecular size between 30 and 50 kDa, which contained 17 cellulases, 6 hemicellulases, 8 pectinases, 5 chitinases, 5 oxidoreductases, and 11 esterases. Afterward, the specific proteins in each treatment were also evaluated to discover critical enzymes. In CK, 9 of 36 unique proteins with signal peptides were identified as cellulases (4), pectinases (1), chitinases (2), and esterases (2). For Cys, only 18 of 106 unique proteins with signal peptides were obtained, in which one cellulase, two hemicellulases, three pectinases, nine esterases, two oxidoreductases, and one transferase were identified.

### Data-quality control of the transcriptome and proteome

**Fig. 4:**
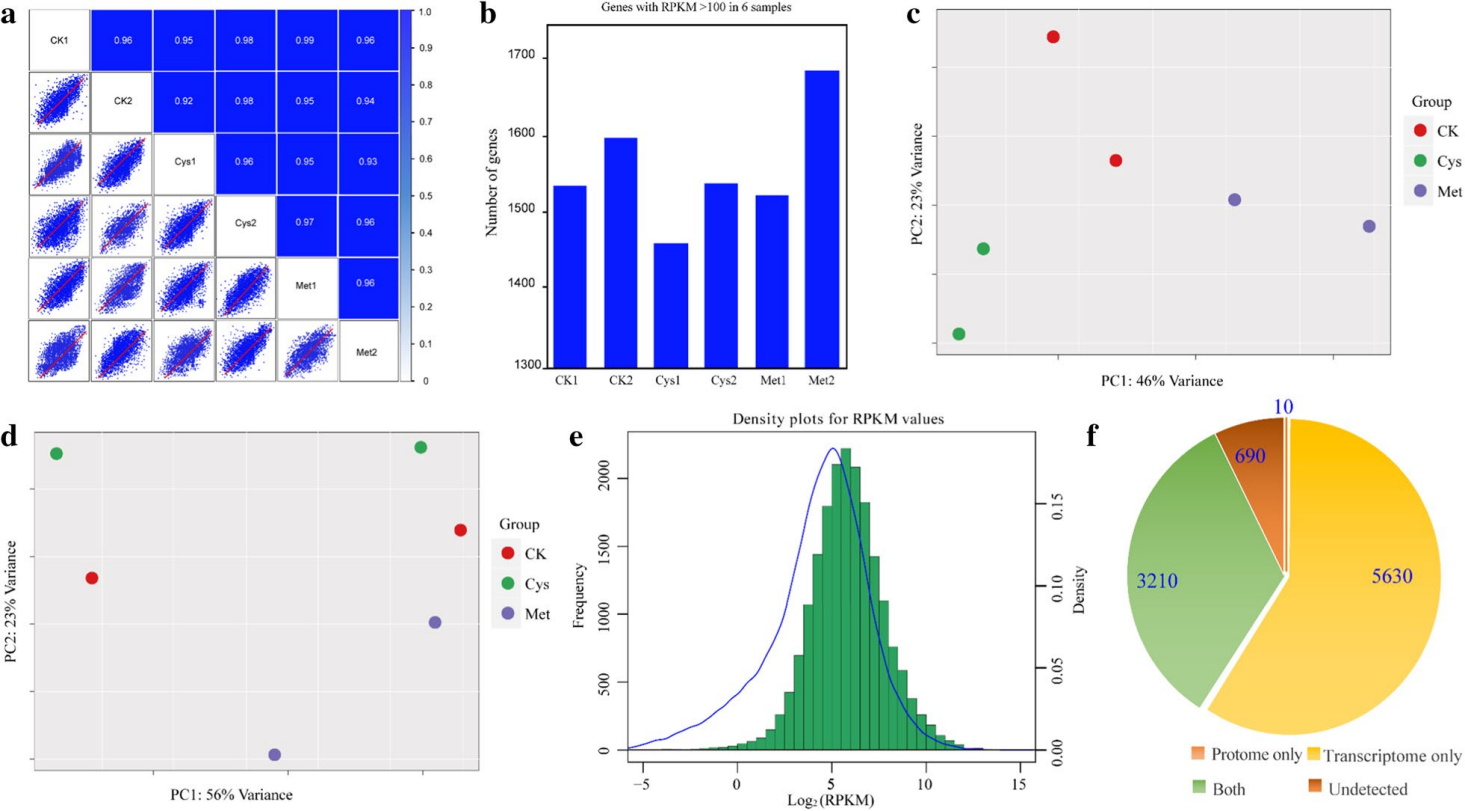
Comparison of proteome and transcriptome data of *A. fumigatus* Z5 with the regulation of different treatments. **a** Pairwise Spearman correlations and scatterplots of − log10 (FPKM values), for the six subjects, show a high consistency of their transcriptomes; **b** a majority of genes with an RPKM value larger than 100 are expressed in all six samples, which showed a high flexibility in whether or not a gene is expressed; **c**–**e** RPKM value distribution of all genes shows one peak, representing highly expressed genes. The genes have been shown to be more likely to be translated into functional proteins; **f** pie chart shows the relationship between the identified proteome and the two omics (proteome and transcriptome)

Isolated mRNA was sequenced at an average depth of 60 million paired-end reads for each sample, and relative abundances in the form of RPKM (reads per kilobase per million mapped reads) values were calculated for each protein-coding gene. The transcriptomes results showed high pairwise correlations (Fig. 4a), and the vast majority of expressed genes (with RPKM > 100) were expressed in all samples (Fig. 4b). An RPKM ≥ 1 has been used as a threshold previously to determine the presence of various protein [18], and it was based on the observation of the distribution of expression abundances. The data obtained in this study also exhibited the same pattern, and the RPKM values of the proteins with supportive reliability (most likely expressed on a protein level) perfectly matched our data (Fig. 4e). Principal component analysis (PCA) was applied to reveal the relationship between different treatments, including CK, Cys, and Met, and the results showed that the differences between the various treatments were significant. Meanwhile, the biological repetitions of different treatments in both the transcriptome and proteome were ideal. These components account for 69% and 79% of the total variability in the transcriptome and proteome, respectively, which exhibited a potential shift between the transcriptome and proteome in samples collected under different environmental conditions (Fig. 4c, d). Both transcriptomic and proteomic analyses were carried out to compare the differences among different treatments, and the results indicated that 5630 genes were exclusively identified at the mRNA level, ten genes were solely found at the protein level, 3210 genes were detected at both levels, and 690 genes were not detected (Fig. 4f). It should be noted that most transcripts not identified at the protein level might be due to the low abundance of related proteins. The proteins not detected in the transcriptome were numerous critical extracellular proteins, which demonstrated that proteomic results could contribute information not accessible at the transcript level and vice versa.

### The description of CAZymes and evaluation of potential lignocellulolytic capabilities of *A. fumigatus* Z5

A total of 269, 21, 55, 96, 69, and 14 genes with a signal peptide were identified as members of the carbohydrate-binding modules (CBM), glycoside hydrolases (GH), auxiliary activities (AA), carbohydrate esterases (CE), glycosyl transferases (GT), and polysaccharide lyases (PL) families, respectively, based on the genomic annotation results. The transcriptome analysis results indicated that there were 269 GH, 21 CBM, 55 AA, 96 GT, 69 CE, and 14 PL genes obtained in the transcriptome. By comparing the genomic information and transcriptome of Z5, the number of proteins identified in each family was the lowest, and 165, 8, 31, 37, 28, and 8 proteins with signal peptides belonged to the GH, CBM, AA, GT, CE, and PL families, respectively (Table 1). The CAZyme transcripts and the proteome results demonstrated that *A. fumigatus* Z5 possessed a considerable capability to degrade lignocellulose by secreting various enzymes. As the major components of extracellular enzymes, several cellulases including, endo-glucanases from GH5, GH7, GH45, GH12, and AA9, cellobiohydrolases from GH6 and AA8, and β-glucosidases from GH1 and GH3, were identified both in the transcriptome and proteome. Significant types of hemicellulases, including arabinoxylanases/glucuronoarabinoxylanases (putative xylanases from GH10 and GH11; β-xylosidases and α-l-arabinofuranosidases from GH43; β-galactosidases from GH2; α-glucuronidases from GH67), glucomannanases/galactoglucomannanases (mannanases and mannosidases from GH92 and GH38; β-galactosidases from GH2), mixed glucanases (β-(1-3,1-4) endo-glucanase from GH16; β-glucanases from GH1 and GH3), and xyloglucanases (xyloglucanases from GH67; α-fucosidase from GH95) were identified. In addition to cellulases and hemicellulases, transcriptome results also showed that Z5 has the capacity to degrade many other substrates, including laminarin (1,3-β-endo-glucanase from GH55; β-glucosidases from GH1 and GH3), starch (α-amylase from GH13), pectin (polygalacturonase from GH28; endo-β-1,4-galactanase from GH53; α-l-rhamnosidase from GH78; pectate lyases from PL3 and PL9; pectin lyase from PL1), chitin (chitinase from GH18 and GH51), and polygalactosamine (endo-α-1,4-polygalactosaminidase from GH114) (Additional file 3: Dataset S2, Additional file 4: Dataset S3, Additional file 5: Dataset S4).

**Table 1:** Identification results of the secretomes of *A. fumigates* Z5 in different treatment

CAZy families	Genome	singalP		Proteome	singalP		Transcriptome	singalP	
		*Y*	*N*		*Y*	*N*		*Y*	*N*
AA	35	16	19	16	8	8	35	16	19
AA/CBM	20	14	6	15	10	5	20	14	6
CBM	19	9	10	7	4	3	19	9	10
CBM/GH	2	1	1	1	0	1	2	1	1
CE	67	32	35	28	14	14	67	32	35
CE/CBM	2	2	0	0	0	0	2	2	0
GH	249	151	98	150	99	51	249	151	98
GH/CBM	18	15	3	14	13	1	18	15	3
GH/GT	2	2	0	1	1	0	2	2	0
GT	96	7	89	37	4	33	96	7	89
PL	14	12	2	8	8	0	14	12	2

*GH* glycoside hydrolases, *AA* auxiliary activities, *GT* glycosyl transferases, *CE* carbohydrate esterases, *PL* polysaccharide lyases, *CBM* carbohydrate-binding modules

RDA analysis was carried out to reveal the correlations between different proteins and various enzyme activities, as shown in Additional file 1: Figure S5, and RDA1 and RDA2 represented a total difference of 97.2% and 2.6%, respectively. It can be seen from the plot that several highly expressed proteins (red line) had a good positive correlation with various enzyme activities (black line). The upregulated beta-glucosidase eglC (A0A0J5PSU6) was positively correlated with beta-glucosidase activity, and the highly expressed endo-1,4-beta-xylanase (A0A0J5Q3N2) was positively correlated with xylanase activity. It is especially necessary to note that the enzymes, including glucanase (A0A0J5Q504), exo-beta-1,3-glucanase (A0A0J5T0L7), and endo-glucanase (A0A0J5T0L7), detected in the proteome, were significantly positively correlated with endo-glucanase activity. Thus, the proteomic analysis results were highly consistent with the corresponding enzyme activity determination results.

### Proteome–wide interaction

**Fig. 5:**
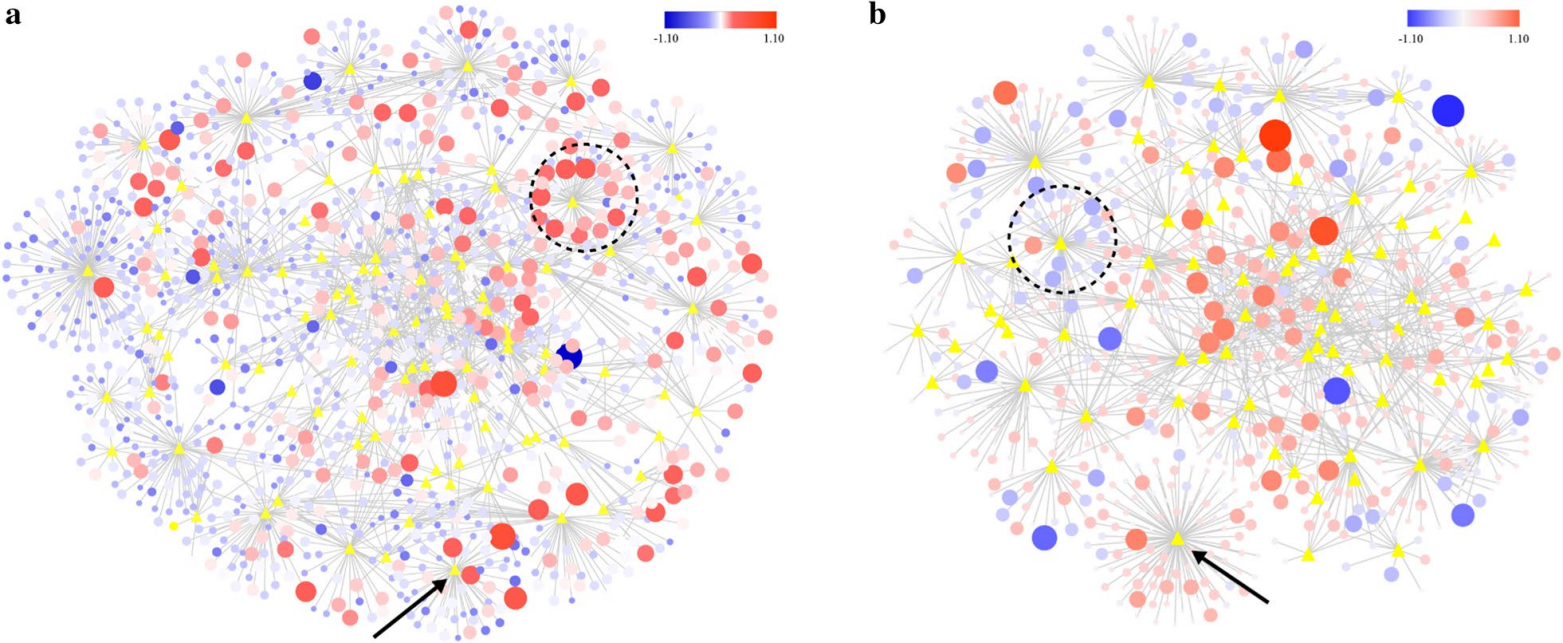
Proteome–wide expression changes on cellulose fermentation visualized as a cytoscape interaction network. Nodes are proteins (circles) or KEGG categories (yellow diamonds); edges are protein interactions defined by KEGG data. Protein node sizes show protein expression (absolute protein expression, APEX). Node colors are expression changes as log2-fold changes. The black dotted circles in **a**, **b** are the starch and sucrose pathways and their associated proteins. The black arrow in **a** indicates the oxidative phosphorylation pathway, and the black arrow in **b** indicates the ribosome pathway

Protein–protein interaction analysis was carried out through cytoscape to reveal the relationship of various proteins involved in different metabolic pathways. The proteome–wide interaction networks of different proteins connected to the KEGG categories (yellow triangles) could be applied to demonstrate the expression level of different proteins and the relationship between the proteins and specific metabolic pathways (Fig. 5). The protein node sizes indicate the expression level of different proteins, while node colors indicate the upregulation (red) or downregulation (blue) of different proteins in the form of log2 FC. Compared to CK, the proteins involved in various metabolic pathways presented different expression levels, and it was even more interesting to find that most of the proteins involved in starch and sucrose metabolism were significantly upregulated, which indicated that the addition of cysteine could improve the lignocellulose degradation capacity of Z5 (Fig. 5a). Figure 5b represents Met, in which the proteins involved in various amino acid metabolism pathways were upregulated significantly, while the proteins in the carbon-related metabolism pathways were downregulated significantly. Simultaneously, we noted that the proteins involved in the ribosome pathway were upregulated in Met, which further illustrated that the addition of methionine promoted amino acid metabolism and inhibited carbon-related metabolism pathways (Additional file 6: Dataset S5).

### KEGG analysis of different treatments

**Fig. 6:**
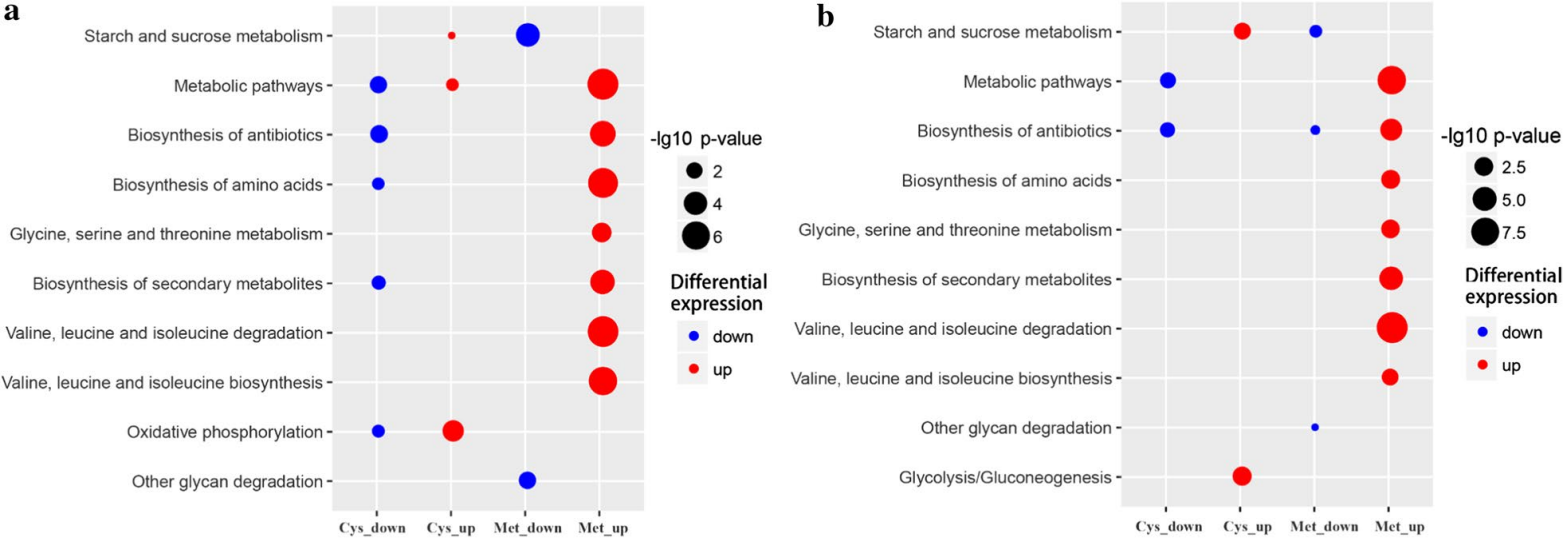
KEGG enrichment of various pathways in different treatments: **a** KEGG enrichment of the proteome in different treatments; **b** KEGG enrichment of the transcriptome in different treatments

The bubble chart was a suitable graphical to display the Kyoto Encyclopedia of Genes and Genomes (KEGG) enrichment analysis results, and the KEGG enrichment was evaluated by − log_10_ *p* value. The higher significant − log_10_ *p* value indicated a more substantial enhancement. The ten most critical enrichment pathways were selected to compare the different metabolism of Z5 under various treatments. Figure 6a shows the most significant enrichment pathways in the proteome, and Fig. 6b shows the most significant enrichment pathways in the transcriptome. The results indicated that starch and sucrose metabolism increased in Cys, while decreasing sharply in Met in the proteome analysis results, which exhibited a similar trend with the results obtained in the transcriptome analysis. The amino acid synthesis and metabolic pathways in Met were mainly enriched, whereas most of these pathways in Cys were downregulated or not changed, which occurred both in the transcriptome and proteome. Similar to other carbon-related metabolism pathways, starch and sucrose metabolism and glycolysis/gluconeogenesis in Cys were upregulated. In addition, many transporters and carbohydrate enzymes, proteins involved in glycolysis and the citric acid cycle (TCA cycle), were also upregulated in Cys. In contrast, most of the same pathways mentioned above were downregulated in Met. Interestingly, the application of methionine in solid medium resulted in the upregulation of methionine metabolism and many other amino acid metabolic pathways, including glycine, serine, and threonine metabolism pathways. Our study found that different pathways could be regulated by these two amino acids (Fig. 6a) (Additional file 7: Dataset S6, Additional file 8: Dataset S7).

**Fig. 7:**
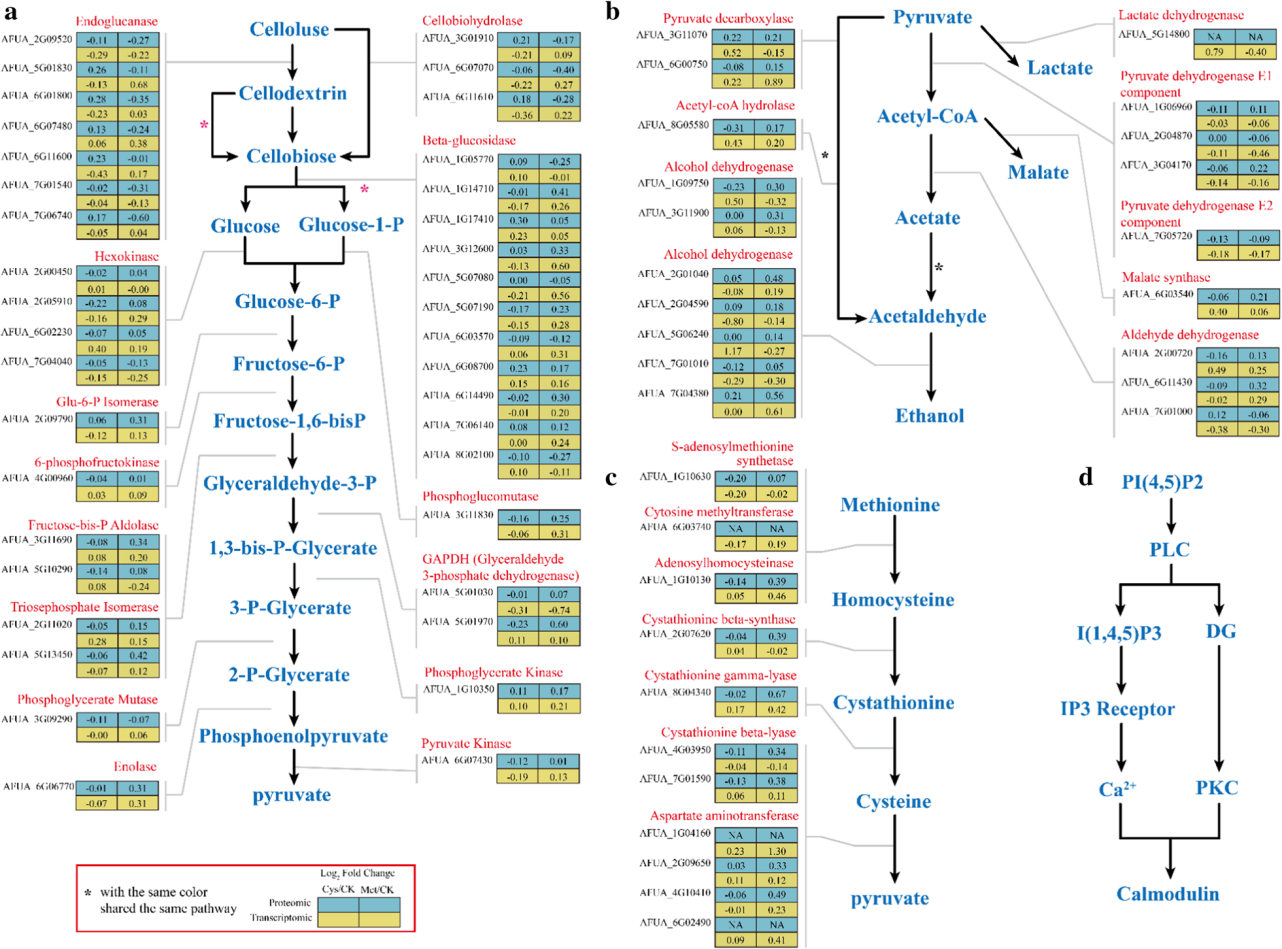
Expression of genes and proteins involved in cellulose breakdown and metabolism pathway during solid-state fermentation. **a** Normalized differential values (log_2_FC) of both the transcriptome and the proteome of reactions involved in the conversion of cellulose to pyruvate; **b** normalized differential values (log_2_FC) of both the transcriptome and the proteome of reactions involved in the conversion of pyruvate to ethanol; **c** normalized differential values (log_2_FC) of both the transcriptome and the proteome of reactions involved in the transformation of methionine to ethanol; **d** upregulation of the calmodulin pathway under the influence of cysteine

The analysis results of the transcriptome and proteome suggested that the celluloses were mainly converted to phosphoenolpyruvate (PEP) via the glycolysis/gluconeogenesis pathway (Fig. 7), and the Log_2_FC value of each protein indicated that most of the cellulose-degrading related enzymes were activated in Cys. During the process of converting cellulose into cellobiose, 11 proteins were found to be upregulated in the proteome, and these proteins contained five extracellular endo-glucanases (AFUA_5G01830, AFUA_6G01800, AFUA_6G07480, AFUA_6G11600, and AFUA_7G06740), two cellobiohydrolases (AFUA_3G01910 and AFUA_6G11610), and four β-glucosidases (AFUA_1G05770, AFUA_1G17410, AFUA_6G08700, and AFUA_7G06140). These enzymes played a crucial role during the cellulose degradation process, and the degrading products could enter the glycolysis metabolism pathway to produce ATP for their life cycle. Compared to Cys, all of these proteins involved in the carbon-related metabolic pathway mentioned above were at deficient levels (Fig. 7a). On the other hand, we focused more on the cysteine and methionine metabolism pathways and TCA cycle. In these two pathways, methionine could be converted to pyruvic acid and then enter the TCA cycle for energy production, during which the *S*-adenosylmethionine synthetase (AFUA_1G10630), adenosylhomocysteinase (AFUA_1G10130), cystathionine gamma-lyase (AFUA_8G04340), cystathionine beta-lyase MetG (AFUA_4G03950), cystathionine gamma-synthase (AFUA_7G01590), aspartate transaminase (AFUA_2G09650), and aspartate aminotransferase (AFUA_4G10410) were upregulated 1.1–1.7 times. Pyruvate decarboxylase PdcA (AFUA_3G11070), pyruvate decarboxylase (AFUA_6G00750), pyruvate dehydrogenase E1 component alpha subunit (AFUA_1G06960), pyruvate dehydrogenase E1 beta subunit PdbA (AFUA_3G04170), aldehyde dehydrogenase (AFUA_2G00720), and aldehyde dehydrogenase Alda (AFUA_6G11430) was detected at very high levels in the TCA cycle, and the up multiplier was 1.1–1.7 times higher. Excitingly, these proteins were also quantified, albeit at deficient levels in Cys (Fig. 7b, c). The results above also demonstrated that most of the methionine entered into the cysteine and methionine metabolism pathways, while cysteine seemed to affect the carbon degradation-related pathways. There were no significant differences in the residual nitrogen content between different treatments (Additional file 1: Figure S4); thus, the possibility that the difference in biomass degradation between different treatments due to the results of metabolic constraints while not the effect of nitrogen content.

In this study, the proteome results showed that calmodulin was significantly upregulated in the treatment of Cys (Fig. 7d). Calmodulin could bind to various proteins and participate in signal transduction in multiple signaling pathways, affecting all the aspects of cell functions. Based on the results mentioned above, a possible conjecture is that cysteine was directly used and then converted to a potential growth factor to stimulate cell growth. We also found that some pathways affecting cell growth appeared to be upregulated, including the mitogen-activated protein kinase (MAPK) signaling pathway and phosphatidylinositol signaling system.

## Discussion

Lignocellulosic plant biomasses mainly consist of three types of polymers: lignin, cellulose, and hemicellulose, which are interlinked in a heteromatrix, and their relative abundance varies mainly depending on the type of biomass [19]. The main components of lignocellulosic biomass are cellulose (40–50%), hemicellulose (20–40%), and lignin (20–30%), as well as a small number of lipids, proteins, pectin, minerals, and soluble sugars [20]. As a major agricultural waste, rice straw contained 41% cellulose and 20% hemicellulose, which are bound to lignin (12%) through hydrogen and covalent bonds [21]. Moreover, rice straw consists of the epidermis, mechanical tissue, primary tissues, and vascular bundles, and the siliceous and cork are combined into a dentate nodular structure [22]. Usually, the cellulose, hemicellulose, and lignin in the untreated rice straw cell walls are intertwined. Therefore, it is difficult for this material to be rapidly and efficiently degraded by ordinary microorganisms secreting various extracellular enzymes.

As shown in Fig. 2, the cell walls of rice straw in CK and Cys were extensively damaged, and the whole straw became very fluffy and porous. The principal reason for such an outcome might be that the degradative enzymes secreted by *A. fumigatus* Z5 can act on the rice straw, resulting in the erosion of the cell wall and the intercellular layer. After weakening or eliminating some of the chemical bonds between the protective layer and lignin and carbohydrates, the cellulose and hemicellulose are quickly degraded by the cellulase and hemicellulase, thus accelerating the decomposition speed of the rice straw. In Met, the secretions of *A. fumigatus* Z5 were inhibited by methionine, and the surface of the rice straw was still very smooth, especially when only a narrow long hole was detected. All the results mentioned above demonstrated that *A. fumigatus Z5* destroyed the lignocellulosic substrates by secreting various extracellular enzymes.

Lignocellulases, especially cellulases and hemicellulases, played a critical role during the lignocellulosic biomass degradation process, and the production of lignocellulose-degrading enzymes was the main bottleneck during bioenergy production. In this study, maximal activities all of the enzymes were obtained in Cys, especially for xylanase activities, while methionine had a significant inhibitory effect on the production of lignocellulases. As a structural unit of a protein, amino acids belong to nitrogen-containing organic compounds, and they have a profound effect on the cellulase synthesis of fungi. Specific side groups of amino acids also regulate the synthesis of various extracellular enzymes. Cristica et al. [23] reported that the addition of glutamic acid and asparagine in growth medium could increase the cellulase and β-xylanase activity of the filamentous fungus *Trichoderma reesei* QM-9414, while the presence of methionine in the medium caused a decrease in enzyme activities [23]. Several amino acids were applied to evaluate the production of cellulases by a brown rot fungus *Fomitopsis* sp. RCK2010, and the results indicated that the maximum CMCase activity was observed in the presence of l-glutamic acid, whereas asparagine, aspartic acid, and cystine comparatively brought about a minor increase in CMCase production. Interestingly, the addition of phenylalanine and methionine could completely inhibit the CMCase production [24], which was consistent with the results obtained in this study. Amino acids also can regulate the synthesis of extracellular enzymes in the other fungi. Vyas et al. [25] found that methionine, asparagine, and tryptophan could promote cellulase synthesis in *Aspergillus terreus*.

Endo-glucanases, exo-glucanases, and β-glucosidases are critical enzymes during the cellulose hydrolysis process, and most fungi secrete these types of enzymes. Fifteen endo-glucanases, four exo-glucanases and six β-glucosidases, several cellobiose dehydrogenases (CDH), and GH enzymes were identified in the extracellular proteins of Z5, which emphasizes the fact that *A. fumigatus* Z5 has considerable potential for cellulose degradation. As an efficient polysaccharide degrader, Z5 encoded 263 glycosyl hydrolase (GH) genes based on the genome annotation results [26], and these enzymes were presumed to be involved in the defense system [27], morphogenetic–morphological processes during fungal development and differentiation [28–30], and mobilization of glucans during energy source and carbon exhaustion [31]. The results showed that the secretomes of *A. fumigatus* Z5 in Cys contained both GPI-anchored and non-GPI-anchored proteins, and partial non-GPI enzymes were involved in the degradation of lignocellulosic material to acquire reducing sugars for growth [29]. The secretomes of *A. fumigatus* Z5 in Cys highlighted the presence of hydrophobin Hyp1 and conidial hydrophobin RodB that played a critical role in mediating contact and communication between the fungus and its environment. Meanwhile, Hsp70 chaperone proteins that play a significant role in stabilizing partially folded proteins and aid in the transmembrane transport of proteins were also identified in this study [32]. Hemicellulose is very heterogeneous and complicated. Hence, its hydrolysis into simpler constituents, including dimers, monomers, and oligomers, requires a broad spectrum of enzymes. Thirty-three hemicellulolytic proteins were identified in the secretome of *A. fumigatus Z5* in Cys, which displayed the greatest abundance of these proteins among all the treatments. Thus, these results also illustrated that cystine could increase the degradation of hemicellulose. Lignin is an amorphous high molecular mass polymer composed of phenylpropane subunits interconnected by massive nonhydrolyzable bonds [33]. *Aspergillus* spp. can transform a broad spectrum of lignin-related aromatic compounds, and species such as *A. japonicus*, *A. niger*, *A. terreus,* and *A. fumigatus*, have been evaluated the ability of metabolizing ^14^C-labeled aromatic compounds [34]. Yang et al. [35] found that *Aspergillus* spp. F-3 isolated from forest soil, possessed a strong capability to degrade alkali lignin. In addition to lignocellulolytic proteins, 24 peptidases and proteases were also identified in Cys using rice straw as a sole carbon source. According to Albenne et al. [36], 84 proteins, which play a significant role in morphogenesis and the formation of β-pleated sheets, are an integral part of the plant cell wall. Thus, the presence of proteins in the plant cell wall and their possible cross-links with carbohydrates themselves could explain the phenomenon that a vast number of peptidases and proteases were secreted by *A. fumigatus* Z5. The formation of sugars and other nutrients caused by the extracellular degradation of biomass is subsequently transported into the cell. Twenty of different transporters were detected in Cys, and various sugar transport pathways were activated during lignocellulose degradation. In addition to degradation of intracellular proteins and nutrient cycling, fungal proteases have been suggested to be essential for cleavage of the CDH flavin-containing protein domain and β-1,4-endo-glucanase activation [37]. Peroxidases could catalyze the degradation lignin, certainly with the cellobiose dehydrogenases (CDH) [38]. Interestingly, a considerable amount of catalase was found in the unique proteins in Cys, while no catalase was detected in Met and CK. Furthermore, catalase–peroxidase was also secreted explicitly in Cys. These results suggested that Z5 could efficiently decompose lignin. The previous studies indicated that some enzymes from GH61 might receive electrons from the action of CDH, which was secreted in concert with GH61 upon cellulose degradation in some fungi. Meanwhile, enzymes from GH61 have been considered to provide electrons for “Fenton chemistry”-based biomass depolymerization during the degradation process [39]. In the case of the deterioration of lignocellulosic substrates, enzymes from GH61 may also get electrons from lignin [39].

It appeared that *A. fumigatus* Z5 initiated strong oxidoreductase and lignin-attacking enzyme expression using rice straw as the sole carbon source. Together with auxiliary oxidoreductases and lignin-attacking, a large number of hemicellulose and cellulose acting GH and CE enzymes were produced after adding cysteine, which would explain why lignocellulosic enzyme activities were most active in Cys. Unlike the Cys treatment, methionine addition was most likely to support the supply of readily metabolized carbohydrates (sugars) for energy and biosynthetic metabolic processes, resulting in the fact that most of the lignocellulosic enzyme activities were significantly inhibited. In the Cys treatment, most of the upregulated proteins were mainly involved in the starch and sucrose metabolism pathway, and five extracellular endo-glucanases (AFUA_5G01830, AFUA_6G01800, AFUA_6G07480, AFUA_6G11600, and AFUA_7G06740), two cellobiohydrolases (AFUA_3G01910 and AFUA_6G11610), and four β-glucosidases (AFUA_1G05770, AFUA_1G17410, AFUA_6G08700, and AFUA_7G06140) that were highly upregulated played a critical role during the degradation of cellulose. Cellulases are the major members of the GH family that catalyze the hydrolysis of β-1,4-glycosidic bonds of cellulose to glucose [7, 40]. The canonical view of the cellulose depolymerization progress is depicted in detail as follows: endo-β-1,4-glucanases (EG, EC 3.2.1.4) randomly hydrolyzed β-1,4-glucosidic linkages primarily in amorphous regions of polymer fibers; cellobiohydrolases (CBH, EC 3.2.1.-) attached to the carbohydrate chains and processively hydrolyzed the disaccharide units from the end of a string without dissociation after each catalytic event; β-glucosidases (EC 3.2.1.21) converted the cellobiose, the primary product of the endo- and exo-glucanase mixture, to glucose [39]. These enzymes had to act synergistically to degrade of cellulose, because the endo-acting enzyme generated new reducing and nonreducing chain ends for the exo-acting enzymes [41]. Meanwhile, highly upregulated enzymes such as cytochrome b-c1, plasma membrane ATPase, cytochrome c oxidase, and NADH-ubiquinone oxidoreductase B14, were involved in the oxidative phosphorylation process, which suggested the possibility that higher dephosphorylation could generate more energy for all secretory enzyme synthesis and cellular processes [42].

As seen from the above, oxidative phosphorylation pathways and carbon metabolism pathways such as starch and sucrose pathways were most active and upregulated in the Cys treatment, which could provide microorganisms with energy for life activities. In the Met treatment, the most active pathways were amino acid biosynthesis and ribosomal pathways, especially the pathways of arginine and proline metabolism; serine, glycine and threonine metabolism; cysteine and methionine metabolism; alanine, aspartate, and glutamate metabolism; and leucine, valine and isoleucine biosynthesis. Moreover, a variety of upregulated aminoacyl-tRNA synthetases including aspartyl tRNA synthetase (A0A0J5SK79, A0A0J5PP87, and A0A0J5PFT4) were detected in the proteome of Z5, and these enzymes could load the correct amino acids into the molecule of the corresponding tRNA. Thus, the tRNA could perform its translation function from the DNA deoxyribonucleotide sequence information of the DNA to the amino acid sequence information of the protein [40]. Aminoacyl-tRNA synthetases that catalyzed the esterification of amino acids to produce aminoacyl-tRNA required for protein biosynthesis were induced in the Met treatment compared to CK. These synthetases are related to the regulation of amino acid biosynthesis and transportation [41]. It has been documented that the upregulation of proteins involved in amino acid biosynthesis, aminoacyl-tRNA synthetase, and ribosomal proteins could enhance the biosynthesis of structural and functional proteins without adding exogenous amino acids, which could also explain the relative increase in proteins involved in glycolysis/gluconeogenesis, starch and sucrose metabolism, the TCA cycle, and other metabolic pathways [39]. Interestingly, this result was consistent with that obtained in the Cys treatment in this study. For the ribosome pathway, ribosomal proteins were one of the abundantly expressed protein types in cells that were detected in high numbers, and the primary function of ribosomal proteins was to organize protein synthesis [42]. In the Cys treatment, only four ribosomal proteins (50S ribosomal protein L12, 50S ribosomal protein L13, 60S acidic ribosomal protein P2, and 60S ribosomal protein L18) were highly upregulated, while almost all the ribosomal proteins involved in the ribosome pathway were upregulated in the Met treatment. Along with these ribosomal proteins, we also identified proteins involved in DNA replication, DNA repair, and translational machinery, including several translation elongation factors and translation initiation factors. The proteins involved in translation and protein biosyntheses, such as isoleucyl-tRNA synthetase (A0A0J5SXS8), elongation factor Tu (A0A0J5PW00), and translation initiation factor IF-2 (A0A0J5PL63, A0A0J5PWI2), were also upregulated. However, protein synthesis is always affected by various environmental factors and nutritional conditions [43]. Interestingly, the Met treatment had a higher upregulation fold than that of the Cys treatment in amino acid anabolism and ribosomal pathways; however, its cellulose utilization capacity was sharply lower than that of Cys. The reason was most likely because methionine was a more natural amino acid for metabolism compared to other nitrogen sources, and it could also enhance various amino acid metabolism pathways in microorganisms. The previous studies showed that the methyl group released from lignin substrates entered the model organism and was used in a large amount for the biosynthesis of methionine in the C1 pathway [43], which was similar to the results obtained in this study. Based on the proteome and transcriptome data, exogenous methionine entered the C1 pathway in the Met treatment (Fig. 7b, c), and strain Z5 could absorb methionine from the medium. The main metabolic pathway of methionine in organisms is to provide various methyl groups through various transmethylation pathways, which could be used as various substances in vivo including methylation of DNA, RNA, or other amino acid biosynthesis processes. Moreover, methionine could be transformed into homocysteine and cysteine and finally produced pyruvate, which participated in the TCA cycle and provided energy to the organism. Through this pathway, the synthesized ATP could satisfy cell growth and reduce the secretion of ligninolytic enzymes. For the Cys treatment, the exogenously added cysteine could be directly absorbed and converted into pyruvic acid to participate in the TCA cycle while reducing the thiol group contained in it, which was the active group of many proteins and enzymes. Many important enzyme activities were related to sulfhydryl groups on cysteine residues. Based on the proteome data, the two signaling pathways of the phosphatidylinositol signaling system and the MAPK-signaling pathway were upregulated. Interestingly, the sho1 and calmodulin pathways were also upregulated, and both of them played an important role in the growth of microbial cells [44, 45]. Moreover, the proteomic data showed that the expression levels of various cellulase-related proteins were also highly upregulated (Fig. 7), which indicated that most of the energy was used for cellulase production in this treatment, and Z5 could better utilize the carbon source in the medium, resulting in the promotion of its growth. A possible function of cysteine for different microorganisms is the stimulation of microbial growth by enhancing signaling pathways such as MAPK-signaling pathways, thereby producing more cellulases for a better utilization of rice straw. In addition, based on the results obtained in this study, there were still some amino acids that could contribute to the promotion or inhibition of cellulase production, which suggested that the use of other N forms by Z5 might not be the result of insufficient intake capacity, but rather metabolic constraints.

In many cases, the response measured at the mRNA level is consistent with the response at the protein level, as illustrated herein by genes involved in glycolysis and starch and sucrose metabolism pathway. In sucrose and starch metabolism, the endo-glucanase (AFUA_2G09520 and AFUA_7G01540), cellobiohydrolase (AFUA_6G07070), and β-glucosidase (AFUA_1G05770, AFUA_1G14710, AFUA_1G17410, AFUA_5G07190, AFUA_6G08700, AFUA_6G14490, and AFUA_7G06140) in the proteome and transcriptome results remained consistent. Hexokinase (AFUA_2G05910, AFUA_7G04040), phosphoglucomutase (AFUA_3G11830), triosephosphate isomerase (AFUA_5G13450), phosphoglycerate mutase (AFUA_3G09290), phosphoglycerate kinase (AFUA_1G10350), enolase (AFUA_6G06770), and pyruvate kinase (AFUA_6G07430) also showed consistency between the two omics in the process of participating in glycolysis. In addition, there was a significant difference between the proteome and the transcriptome results, most notably regarding the genes involved in energy metabolism. Proteins such as acetyl-coA hydrolase (AFUA_8G05580), malate synthase (AFUA_6G03540), and aldehyde dehydrogenase (AFUA_2G00720, AFUA_7G01000) showed significant differences between the transcriptome and proteome results. The differences in mRNA and proteins between these genes indicated the post-transcriptional control mechanisms of these proteins involving, for example, protein half-life and thereby accumulation of the protein or protein modification, these data alone were not sufficient to explain the exact regulatory mechanisms [46], but the data do provide direction for more specific targeted experiments. Anyhow, this study could improve our understanding of the mechanisms of protein regulation in different pathways.

A large number of cellulosic wastes have been discarded or used inefficiently due to the high costs of utilization processes [47], while the conversion of lignocellulosic biomass into soluble sugars is the primary bottleneck of these processes and depends mainly on the production of various efficient lignocellulolytic enzymes [48]. In this study, the production of lignocellulosic enzymes of *A. fumigatus* Z5 could be significantly increased by supplying 0.2% cysteine to the culture medium, and the mechanisms were also revealed through transcriptome and proteome methods. Overall, the findings extend our knowledge of the transformation of various lignocellulosic materials, with anticipated benefits for the development of the bioenergy industry. However, caution must be used in that the effects on the production of various lignocellulosic enzymes by various amino acids are very different from each other. Shikha et al. reported that methionine, tryptophan, glycine, and valine could stimulate laccase production by *Cyathus bulleri*, while cysteine monohydrochloride completely inhibited enzyme production [49], which was contrary to the results obtained in this study. Thus, before different amino acids are used to promote lignocellulosic biomass utilization, detailed parameters, including the types and contents of different amino acids, should be studied.

## Conclusions

This study demonstrated that cysteine could promote the growth of *A. fumigatus* Z5 and enzyme productions, while methionine had a significant inhibition effect. The main reason for these results was that Z5 used different ways to utilize exogenous amino acids to adapt to the external environment, and the differences in growth and enzyme activities among different treatments might be due to metabolic constraints. All of the results obtained above enhance our understanding of the physiology and metabolism of *A. fumigatus* Z5, and contribute to increasing the knowledge of fungal genetics and lignocellulose bioconversion pathways.

## Experimental procedures

### Culture medium, inoculation, and strain growth conditions

Rice straw was obtained from local farmland, and it was chopped into small pieces with a length of 1–2 cm after air drying and then ground into smaller particles in a Chinese herbal medicine mill. The efficient lignocellulosic decomposing strain Z5 was isolated from the compost and identified as *A. fumigatu*s in a previous report [50], and it was maintained on a potato dextrose agar (PDA) medium slant at 4 °C. The strain was grown on PDA medium for conidia production and incubated at 50 °C under static cultivation conditions for 7 days. Then, the conidia were harvested by washing the plate with 10 mL of sterile ddH_2_O followed by removal of mycelia by filtration through four layers of gauze. The conidia were resuspended, and the concentration was adjusted to 1 × 10^6^ conidia mL^−1^. The resulting conidial suspension was used as the primary inoculum for further experiments.

### Effects of different pure amino acids on the production of lignocellulose secreted by *A. fumigatus* Z5

Mandels’ salt solution without organic components (1.4 g L^−1^ (NH4)_2_SO_4_, 2.0 g L^−1^ KH_2_PO_4_, 0.3 g L^−1^ CaCl_2_, 0.3 g L^−1^ MgSO_4_, 5 mg L^−1^ FeSO_4_·7H_2_O, 20 mg L^−1^ CoCl_2_, 1.6 mg L^−1^ MnSO_4_ and 1.4 mg L^−1^ ZnSO_4_) [51] supplemented with 2% (w/v) rice straw was used for cellulase production under liquid-state fermentation, and stock solutions of 16 different pure amino acids were filter sterilized and added into different sterilized Erlenmeyer flasks at a final concentration of 0.2% (w/v) [49]. The amino acids applied in this study mainly included aspartic acid, threonine, serine, glutamate, glycine, alanine, cysteine, valine, methionine, isoleucine, leucine, tyrosine, phenylalanine, lysine, histidine, and proline, which could also be used as a nitrogen source for *A. fumigatus* Z5. The nitrogen content of the various culture media was balanced by ammonium sulfate. Culture medium with ammonium sulfate as the sole nitrogen source was regarded as the control treatments. Two hundred milliliters of litter media containing 200 mL of Mandels’ salt solution with 4 g of milled dry rice straw in 500 mL Erlenmeyer flasks were autoclaved for 30 min at 115 °C. A 1% (v/w) fresh conidial suspension (1 × 10^7^ conidia mL^−1^) of *A. fumigatus* Z5 was inoculated into the flasks, and all flasks were cultured at 37 °C at 170 rpm until the enzyme assays [4].

### Cellulase production under solid-state fermentation

Solid-state fermentation was carried out in 250 mL Erlenmeyer flasks, each with 9.0 g of rice straw with Mandels’ salt solution medium to attain a moisture content of 70%. The containers were sterilized by autoclaving at 121 °C, after which 1% (v/v) of fresh conidia suspension was inoculated into the sterilized solid medium. Two amino acids (cysteine and methionine) chosen based on the previous analysis results were used as additives, and all treatments were supplied with an equal quantity of nitrogen. The initial pH of the medium was adjusted to 5.0 before sterilization, and the nitrogen content of the solid medium was balanced by solid ammonium sulfate. Five grams of fermented substrates were aseptically taken from flasks after 4 days of cultivation, and then suspended in 50 mL of deionized water and shaken gently for 1 h [52]. The substrates and fungal biomass were removed by centrifugation (10,000*g* for 10 min at 4 °C) and further clarified by filtration through a 0.45 μm membrane (Beyotime, China). The clear supernatants were used as the crude enzymes in the subsequent experiments, and the rest of the fermentation products including the substrates and fungal hypha were used for the extraction of proteins [32].

### Evaluation of the degradation degree of rice straw by electron microscopy

The growth of fungal mycelia and the degree of straw decay in solid fermentation samples inoculated with *A. fumigatus* Z5 spores for 4 days were observed by scanning electron microscopy. The rice straw in the solid fermentation sample was first collected, cut into a certain size with a sharp blade and washed [52], and then fixed with a fixing solution (2.5% glutaraldehyde), followed by dehydration using increasing concentrations of ethanol (from 20 to 98%, v/v) and acetone (from 30 to 90%, v/v); finally, the critical point dryer (HCP-2, Hitachi High-Technologies Corporation, Japan) was used to critically dry the sample and a layer of nanometer thick Au/Pd alloy layer was sprayed using a Cressington 208 HR high-resolution sputter coater (Cressington, UK). Imaging can be performed using the Hitachi S-4800 FE-SEM (Hitachi, Japan) after sample preparation.

### Enzyme assay

Distilled water was added to the solid sample cultured for 4 days at (1:10, w/v), and the supernatant was centrifuged (10,000×*g* for 10 min at 4 °C) to obtain a crude enzyme solution, and the crude enzyme solution was stored at 4 °C for the subsequent experiments. Endo-glucanase activity and xylanase activity were determined by Miller’s dinitrosalicylic acid (DNS) method using sodium carboxymethylcellulose [53] and xylan [54] as substrates, respectively. Twenty microliters of the crude enzyme were mixed with 0.5 mL of 0.5% (w/v) of the corresponding substrate, 50 mM sodium acetate–acetate buffer (pH 5.0) and 0.48 mL of distilled water were incubated at 50 °C for 20 min, and 1 mL of DNS was added, followed by boiling water for 5 min. Moreover, read the color of the color developed at 520 nm. One enzyme activity unit is defined as the amount of enzyme required to release 1 μmol of reducing sugar per minute under the above-described measurement conditions. The chromogenic substrate β-nitrophenyl-β-d-celloglucoside (pNPC) (Sigma, USA) [55] and β-nitrophenyl-β-d-glucopyranoside (pNPG) (Sigma, USA) were separately through a trace amount. The exo-glucanase activity and β-glucosidase activity were measured by the titration plate method [56]. Ten microliters of the crude enzyme were mixed with 25 μL of 200 mM sodium acetate buffer (pH 5.0), 25 μL of 5 mM of the corresponding substrate, and 40 μL of distilled water. The plate was incubated at 50 °C for 10 min, the reaction was stopped by the addition of 100 μL of 1 M Na_2_CO_3_ solution, and the color of the developed color was read at 402 nm. One enzyme activity unit was defined as the amount of enzyme required to release 1 μmol of pNP per minute under the above assay conditions.

### Total RNA isolation, sequencing, and functional annotation analyses

RNA extraction was carried out using RNA extraction kit according to the manufacturer’s instructions (QIAGEN, Germany). RNase-free DNase (QIAGEN, Germany) was used to remove DNA contamination during RNA extraction. The extracted RNA was measured using a NanoDrop spectrophotometer (ND-1000, NanoDrop Technologies, Wilmington, USA) and an Experion Automated electrophoresis system (Bio-RAD, Mississauga, Canada) was used to assess RNA integrity, and finally, RNA sequencing from high-quality RNA samples (RIN > 7) using the Illumina HiSeq2000 platform. According to the RNA-seq analysis method of Borin et al. [57], the RNA-sequenced data were filtered using the AlienTrimmer software [58] after size filtering (minimum 40 bp) and quality selection (*Q* > 20), the screened data were aligned with the *A. fumigatus* Z5 genome available in the UniProt database (A0A0J5Q558_ASPFM, 9540 gene models) using TopHat2 (http://ccb.jhu.edu/software/tophat/index.shtml). RSEM software (RSEM v1.1.17 http://deweylab.github.io/RSEM/) and EBSeq (http://www.bioconductor.org/packages/devel/bioc/html/EBSeq.html, R package version 1.21.0) were used to predict the expression levels of various genes and analyze the differentially expressed genes between the treatment groups. In the differential expression analysis, the Benjamini and Hochberg method was used to correct the *p* value of the original hypothesis (*p* value) [59], and the significant difference in gene expression was determined using the corrected *p* value < 0.05 and log_2_ fold change (FC) ≥ 2. The topGO software (https://bioconductor.org/packages/release/bioc/html/topGO.html, R package version 2.32.0) was used to analyze the enrichment of differentially expressed genes annotated into the GO database. The CAZymes category including GH, CE, PL, and AA genes is predicted by CAZy (http://www.cazy.org/), and the grouping of these different categories is based on functional information.

### Protein identification by peptide LC–MS/MS

Proteins were extracted from solid fermentation products after 4 days of inoculation using the NoviPure™ Soil Protein Extraction kit (Mobio, 30000-20) according to the manufacturer’s protocol. The extracted proteins were redissolved in 100 μL of 100 mM triethylammonium bicarbonate (TEAB) under sonification and then incubated in boiling water for 10 min to denature the proteinase. Protein concentration was determined using the Micro BCA protein assay kit (Beyotime, China). Two hundred micrograms of extracellular proteins were taken for reductive alkylation and purified using TCA–acetone precipitation. The pellet was resuspended by 100 μL of 100 mM TEAB and then digested with trypsin (Promega, Madison, WI) overnight.

The digestion of protein was achieved by adding DTT at the final concentration of 10 mM, and then IAM at 55 mM, and finally trypsin at 1 μg for about 8–16 h. The digested polypeptide was labeled with Pierce TMT 6-plex isotope mass labeling kit (Thermo-Fisher Scientific, Rockford, IL), and the labels were listed as follows: CK1, 126; CK2, 127; Cys1, 128; Cys2, 129; Met1, 130. The labeled peptide samples were merged and dehydrated using Waters Sep-Pak C18 SPE column. The dehydrated and desalted peptide was dissolved in 15 μL loading buffer (0.1% formic acid, 3% acetonitrile). Finally, the eluent was lyophilized in a vacuum concentrator before RPLC grading at high pH. The merged polypeptide peptide was dissolved in buffer A (20 mM ammonium formate in water, pH 10.0), and then separated at high pH using Acquity UPLC system (Waters Corporation, Milford, MA) and reversed-phase column (XBridge C18 column, 2.1 mm × 150 mm, 3.5 μm, 300 Å, Waters Corporation, Milford, MA) with a linear gradient of 5 to 35% of buffer B (20 mM ammonium formate in 90% ACN, pH 10.0, adjusted with ammonium hydroxide) for approximately 40 min to achieve fractional separation. The column velocity is kept at 200 μL min^−1^ and the column temperature was kept at room temperature. A total of 15 different fractions were collected and each fraction was dried in a vacuum concentrator for the subsequent experiments. Separation by nanoLC after suspending different fractions of the preceding step in 30 μL solvent C (water with 0.1% formic acid) was carried out, and the separated products were tested by quadrupole-Orbitrap mass spectrometer (Q-Executives) (Thermo Fisher Scientific, Bremen, Germany) equipped with an on-line spray ion source. Five microliters of samples were loaded on the column (Thermo Scientific Acclaim PepMap C18, 100 μm × 2 cm), and the flow rate was kept at 10 μL min^−1^ for about 3 min. Then, the samples were separated on the analytical column (Acclaim PepMap C18, 75 μm × 15 cm). The linear gradient was set as 2–40% with buffer D (ACN with 0.1% formic acid) for about 100 min. Finally, the column flow rate was maintained at 300 nL min^−1^ and the column temperature was maintained at 40 °C under the initial conditions. Meanwhile, the electrospray voltage was kept at 1.9 kV versus the inlet of the mass spectrometer. The polypeptides were analyzed on an LC–MS/MS instrument (ekspertTM nanoCL; AB Sciex Triple TOF 5600-plus), and the mass spectrometry data of different treatments were automatically collected.

### Protein quantitation and functional prediction of the proteome

MS/MS data analysis was performed using Mascot Distiller software (Matrix Science, London, UK; version 2.5.1, http://www.matrixscience.com) based on the *A. fumigatus* Z5 database (https://www.UniProt.org/, 201605, 9655 entries) [60]. Scaffold Q+ (Proteome Software Inc., Portland, OR, version Scaffold_4.5.3) was used to identify and quantify the peptides and proteins in different treatments. The protein probability was specified by the Protein Prophet algorithm [61]. If a peptide with an FDR was less than 1.0% which was obtained by Scaffold Local FDR algorithm with a probability of more than 78.0%, and then, the peptide identification was used. Statistical analysis of the relative labeled mass spectrometry data of complex samples by variance analysis [62], normalization of intensity repeats (crossing samples and spectra), and logarithmic transformation of different processed spectral data, matching to multiple proteins. Spectral data were trimmed and then weighted using an adaptive intensity-weighting algorithm. The spectra (61758 of 62178) at a given threshold in this study were included in the quantification, and all differentially expressed proteins were determined by the Mann–Whitney test (*p* < 0.05) and a fold change of 1.3-fold.

### Bioinformatics analysis

Annotation of Gene Ontology (GO) was carried out to select the differentially expressed genes or proteins (DEGPs) using Blast2GO software (http://www.geneontology.org). The significant ontologies for GO annotation of DEGPs contained molecular function, biological process, and cellular component. GO enrichment analysis was performed based on all GO terms that were significantly enriched by the DEGPs. For each GO term, the number of genes or proteins was calculated before the hypergeometric test to obtain the significantly enriched GO terms by the input list of DEGPs [63]. Functional interpretation of differentially expressed genes or proteins was performed using KEGG Orthology-Based Annotation System 2.0 (KOBAS) [64]. The *Aspergillus fumigatu*s database was chosen as the backend database. Fisher’s exact test *p* < 0.05 was selected as the threshold of significant change functions and KEGG pathways.

## Additional files

**Additional file 1.** Supplementary figures and tables.

**Additional file 2: Dataset S1.** Identification of the secretomics from *A. fumigatus* Z5 in different treatments.

**Additional file 3: Dataset S2.** Identification of the total proteome from *A. fumigatus* Z5 in different treatments.

**Additional file 4: Dataset S3.** Quantification of the total proteome from *A. fumigatus* Z5 in different treatments.

**Additional file 5: Dataset S4.** Proteins of different families associated with cellulose degradation identified both in the proteome and transcriptome.

**Additional file 6: Dataset S5.** Protein and pathway data required for interactive mapping.

**Additional file 7: Dataset S6.** KEGG and GO analysis results of the total proteome from *A. fumigatus* Z5 in different treatments.

**Additional file 8: Dataset S7.** KEGG and GO analysis results of the transcriptome of *A. fumigatus* Z5 in different treatments.

## Abbreviations

SSF: solid-state fermentation
SmF: submerged fermentation
EG: endo-glucanase
CBH: cellobiohydrolase
pNPC: β-nitrophenyl-β-d-cellobioside
pNPG: β-nitrophenyl-β-d-glucopyranoside
GH: glycoside hydrolases
AA: auxiliary activities
GT: glycosyl transferases
CE: carbohydrate esterases
PL: polysaccharide lyases
CBM: carbohydrate-binding modules
CMC-Na: carboxymethylcellulose sodium
