# Supplementary material for: Effects of amino acids on the lignocellulose degradation by Aspergillus fumigatus Z5: insights into performance, transcriptional, and proteomic profiles
Source: Biotechnol Biofuels. 2019 Jan 4;12:4. doi: 10.1186/s13068-018-1350-2 (PMC6318881; doi:10.1186/s13068-018-1350-2)
Supplement: Supplementary file 1 — Additional file 1. Supplementary figures and tables. [file 13068_2018_1350_MOESM1_ESM.docx]

**Additional data for**

**Effects of amino acids on the lignocellulose degradation of *Aspergillus fumigatus* Z5: Insights into performance, transcriptional and proteomic profiles**

Jiaxi MIAO^1,2^, Mengmeng WANG^1,2^, Lei MA^1,2^, Tuo LI^1,2^, Qiwei HUANG^1,2^, Dongyang LIU^1,2^^[[1]](#footnote-1)^*, Qirong SHEN^1,2^

1 Jiangsu Key Lab for Organic Solid Waste Utilization, Nanjing Agricultural University, Nanjing, 210095, China

2 Jiangsu Collaborative Innovation Center for Solid Organic Waste Resource Utilization

**Running title:** Transcriptome and proteome combination analysis of *A. fumigatus* Z5 under the regulation of various amino acids during lignocellulose degradation process


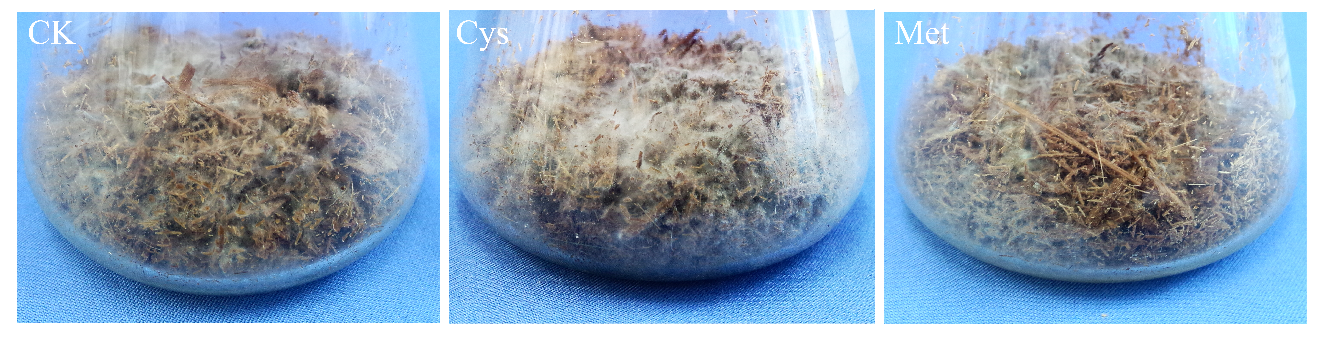


**Figure S1 The growth of *A. fumigatus* Z5 with the regulation of cysteine and methionine in solid-state fermentation.** Cys indicated the treatment adding 0.2% (w/w) cysteine in the medium under solid state fermentation; Met was the treatment adding 0.2% (w/w) methionine in the medium under solid state fermentation; CK was the treatment that ammonium sulfate was used as the sole nitrogen source.

**
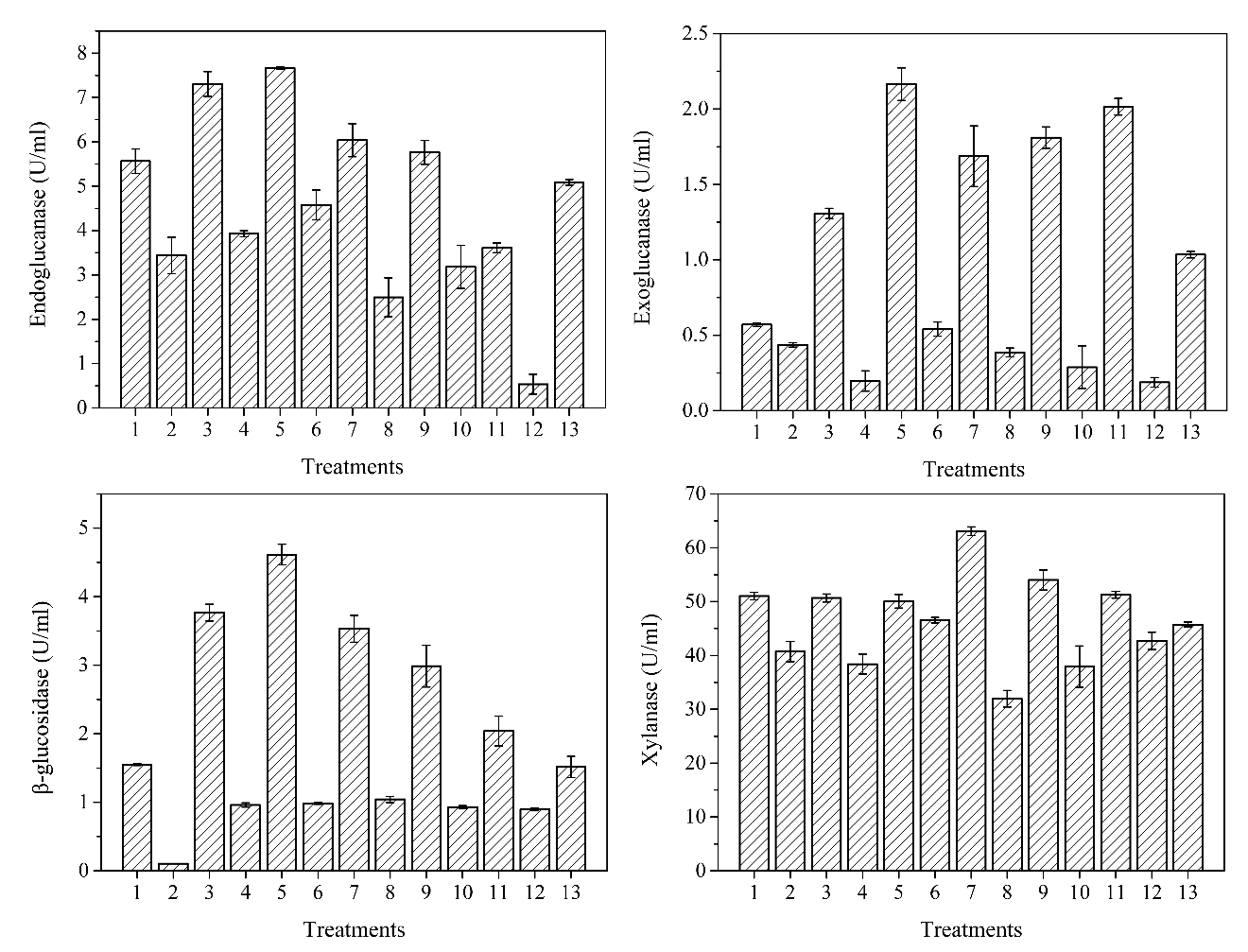
**

**Figure S2 The cellulosic enzyme activities in different treatments with different concentration of amino acids.** 1, 0.05g/L Cys; 2, 0.05g/L Met; 3, 0.10g/L Cys; 4, 0.10g/L Met; 5, 0.15g/L Cys; 6, 0.15g/L Met; 7, 0.20g/L Cys; 8, 0.20g/L Met; 9, 0.25g/L Cys; 10, 0.25g/L Met; 11, 0.30g/L Cys; 12, 0.30g/L Met; 13. CK.


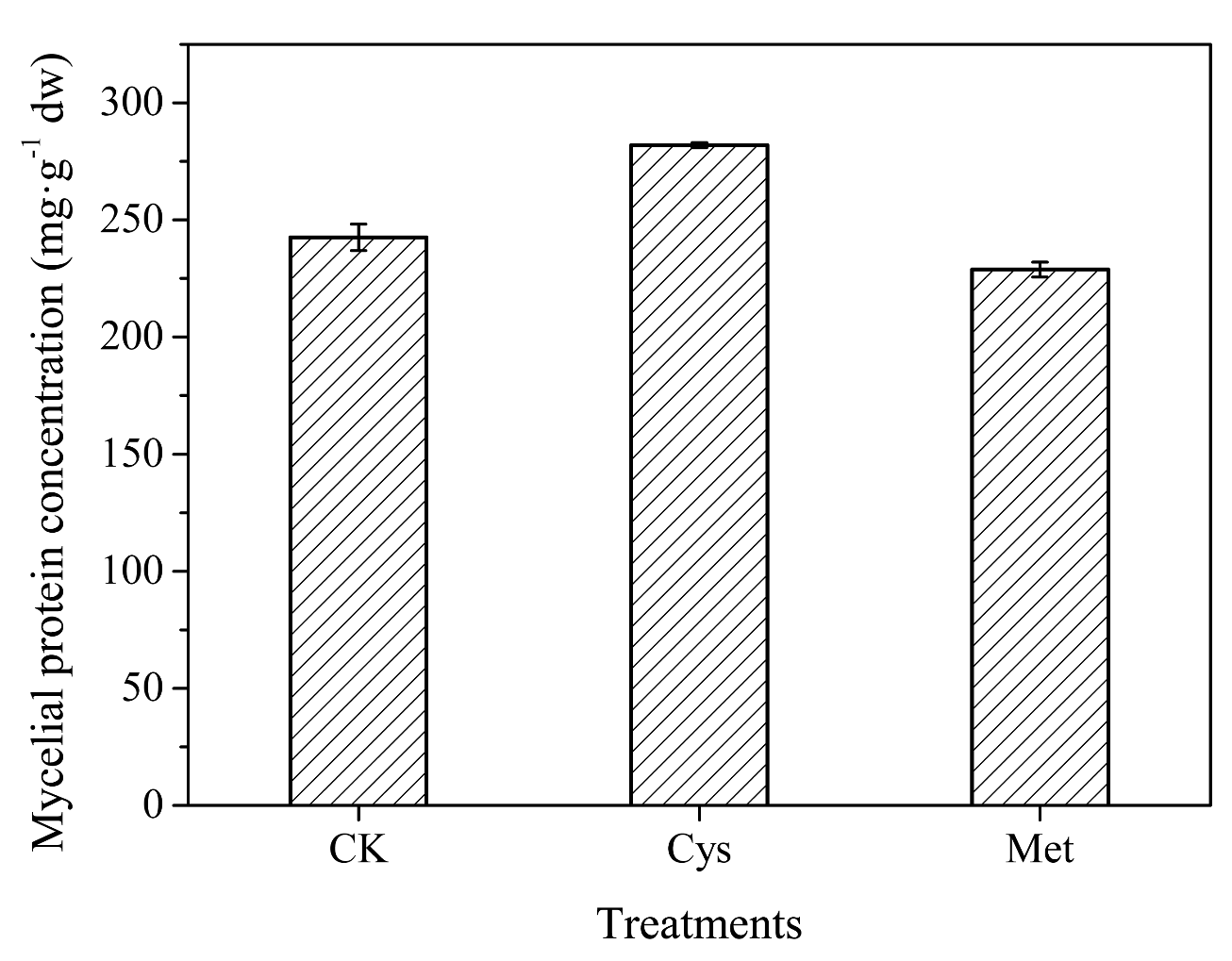


**Figure S3 The content of mycelial protein concentration in different treatments.** Cys indicated the treatment adding 0.2% (w/w) cysteine in the medium under solid state fermentation; Met was the treatment adding 0.2% (w/w) methionine in the medium under solid state fermentation; CK was the treatment that ammonium sulfate was used as the sole nitrogen source.


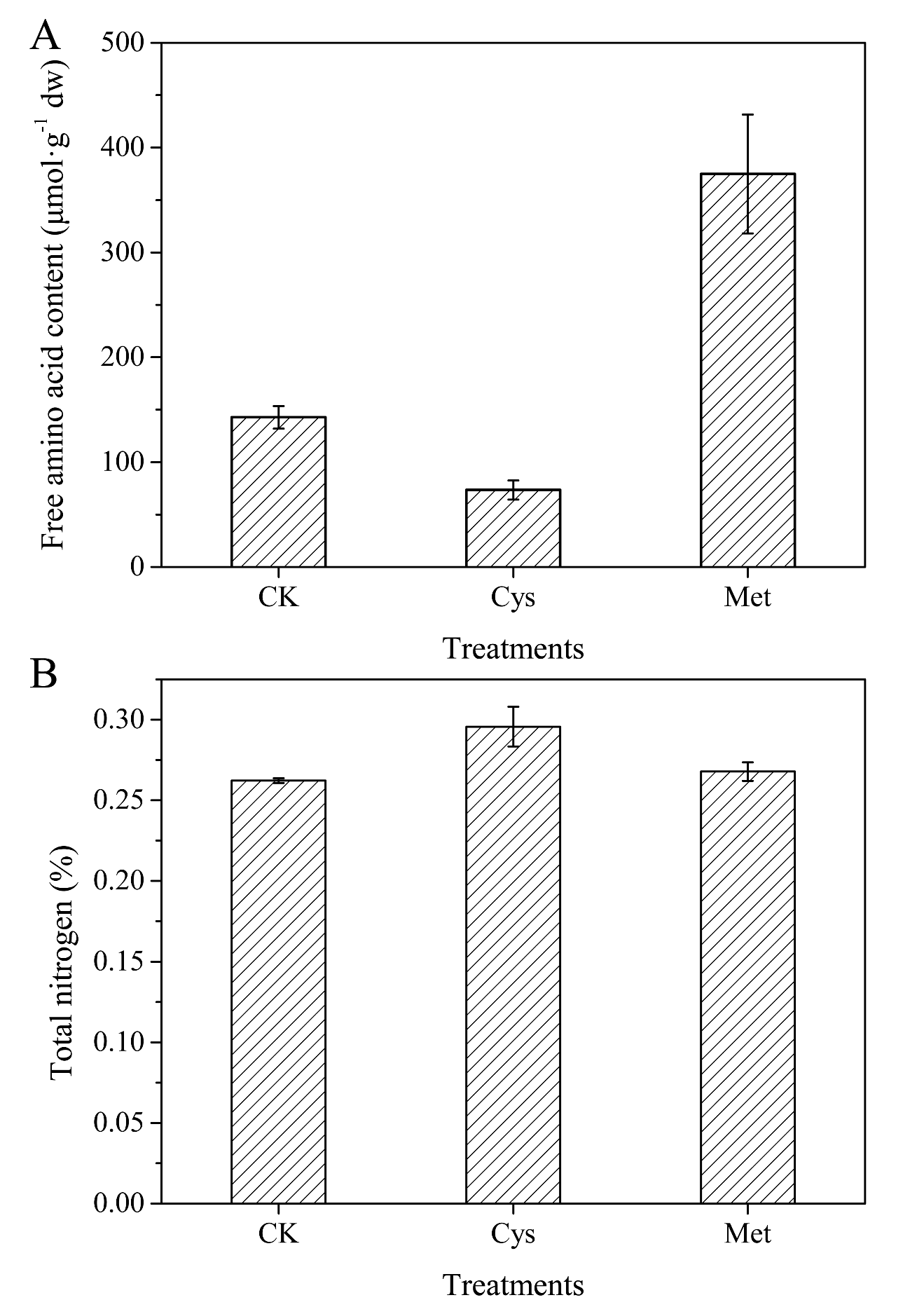
**Figure S4 The contents of free amino acids and total nitrogen in different treatments.** A was the content of free amino acids in different treatments; B was the content of total nitrogen in different treatments; Cys indicated the treatment adding 0.2% (w/w) cysteine in the medium under solid state fermentation; Met was the treatment adding 0.2% (w/w) methionine in the medium under solid state fermentation; CK was the treatment that ammonium sulfate was used as the sole nitrogen source.


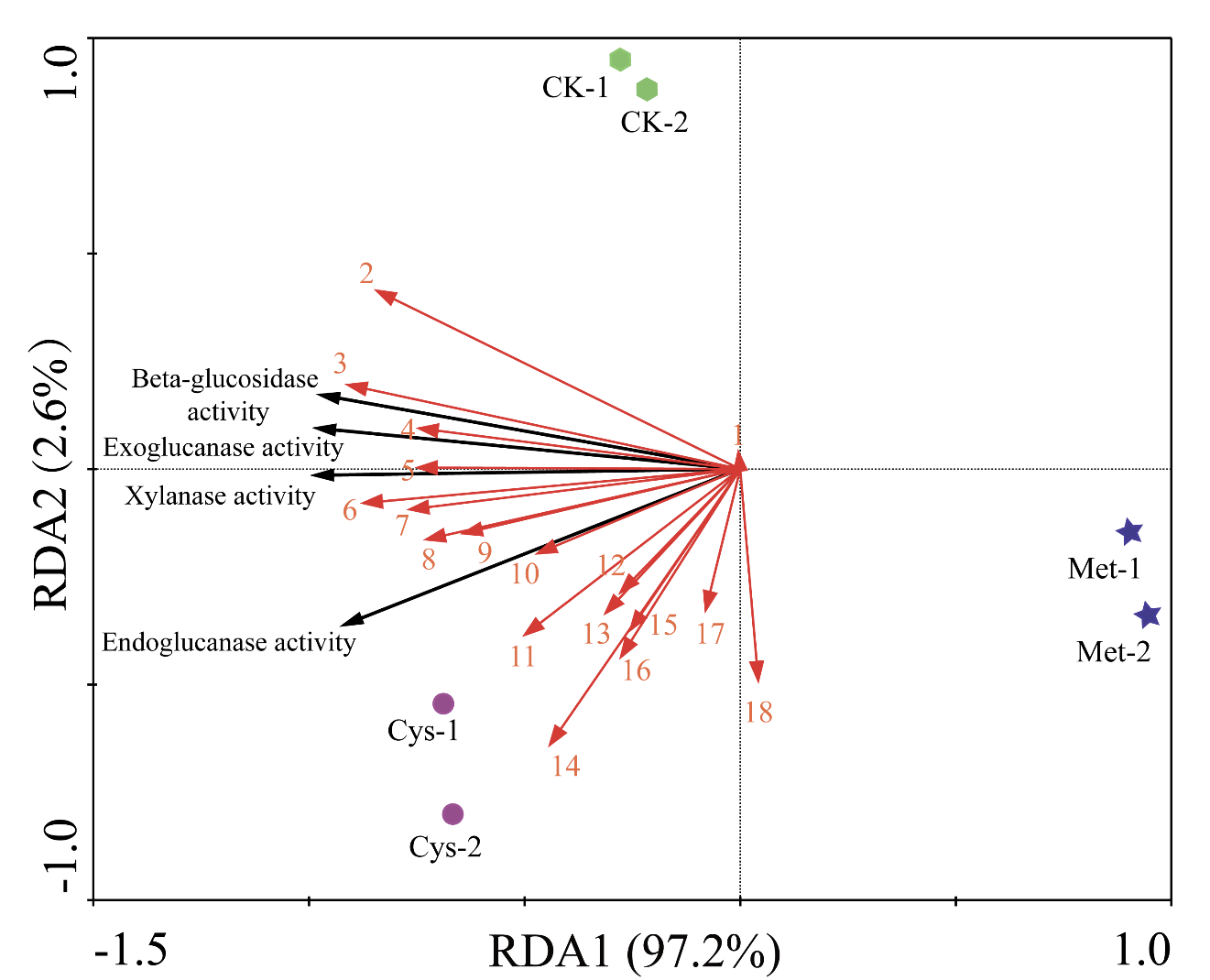


**Figure S5 Plot of the first and second principal components (PCs) of various enzymes activities and the major up-regulated proteins during the biodegradation process.** The percentage of variation explained by each PC is indicated between brackets. 1, beta-xylanase; 2, glucanase; 3, beta-glucosidase eglC; 4, endo-1, 4-beta-xylanase, 5, exo-1, 3(4)-beta-glucanase; 6, glucanase; 7, glucanase; 8, glucanase; 9, exo-beta-1, 3-glucanase; 10, endoglucanase; 11, exo-beta-1, 3-glucanase; 12, beta-xylanase; 13, endo-beta-1, 4-glucanase A; 14, beta-glucosidase M; 15, exo-beta-1, 3-glucanase Exg0; 16, endo-1, 4-beta-xylanase; 17, endo-1, 4-beta-xylanase; 18, beta-glucosidase.

Table S1 the identification results of the secretomes of *A. fumigates* Z5 in different treatments by using rice straw as a sole carbon source

| Protein name | Protein ID | Coverage | Unique Peptide | Uniq Spectrum | Mass | family | Enzyme | Signal | Identified by |
| --- | --- | --- | --- | --- | --- | --- | --- | --- | --- |
| mycelial catalase Cat1 | XP_748550.1 | 0.5179 | 30 | 141 | 79909.7 | catalase like | Oxidoreductase | Y | CK; Cys |
| cellobiose dehydrogenase | XP_756097.1 | 0.4509 | 42 | 1334 | 85542.8 | CDH | Oxidoreductase | Y | Met; CK; Cys |
| alpha-galactosidase | XP_753271.1 | 0.0301 | 1 | 3 | 58990.1 | GH27 | Cellulase | Y | CK |
| endo-1,3(4)-beta-glucanase | XP_751328.1 | 0.0737 | 1 | 3 | 30668.9 | GH16 | Cellulase | Y | CK |
| cell wall glucanase | XP_750740.1 | 0.1 | 2 | 5 | 47104.7 | GH16 | Cellulase | Y | CK |
| extracellular endoglucanase/cellulase | XP_755286.1 | 0.0252 | 1 | 1 | 50811.4 | GH5 | Cellulase | Y | CK |
| beta-glucosidase | XP_746996.1 | 0.139 | 9 | 37 | 87099 | GH3 | Cellulase | Y | CK; Cys |
| exo-beta-1,3-glucanase | XP_754859.1 | 0.0828 | 5 | 18 | 86725.5 | PL 3 | Cellulase | Y | Met; CK |
| endoglucanase | XP_750640.1 | 0.0948 | 2 | 3 | 33439.5 | GH5 | Cellulase | Y | Met; CK |
| endoglucanase | XP_748715.1 | 0.242 | 3 | 14 | 23539.3 | GH5 | Cellulase | Y | Met; CK |
| GPI anchored endo-1,3(4)-beta-glucanase | XP_755769.1 | 0.1028 | 4 | 47 | 65992.4 | GH16 | Cellulase | Y | Met; CK |
| cell wall glucanase (Utr2) | XP_749447.1 | 0.1625 | 8 | 57 | 46709.9 | GH16 | Cellulase | Y | Met; CK |
| extracellular glycosyl hydrolase/cellulase | XP_749229.1 | 0.6364 | 31 | 1060 | 42924.4 | GH62 | Cellulase | Y | Met; CK; Cys |
| exo-beta-1,3-glucanase Exg0 | XP_752815.1 | 0.2133 | 16 | 142 | 100620.9 | PL 3 | Cellulase | Y | Met; CK; Cys |
| endoglucanase | XP_750222.1 | 0.0966 | 2 | 14 | 25313.9 | GH12 | Cellulase | Y | Met; CK; Cys |
| extracellular cellulase CelA/allergen Asp F7-like | XP_753833.1 | 0.2064 | 5 | 11 | 35575.2 | GH | Cellulase | Y | Met; CK; Cys |
| glycosyl hydrolase | XP_749205.1 | 0.2373 | 5 | 21 | 40880.8 | GH76 | Cellulase | Y | Met; CK; Cys |
| extracellular cell wall glucanase Crf1/allergen Asp F9 | XP_752985.1 | 0.3646 | 13 | 306 | 40283.5 | GH16 | Cellulase | Y | Met; CK; Cys |
| beta glucosidase | XP_750759.1 | 0.2511 | 15 | 70 | 94947.5 | GH3 | Cellulase | Y | Met; CK; Cys |
| exo-beta-1,3-glucanase | XP_751203.1 | 0.2687 | 12 | 107 | 84182.9 | PL 3 | Cellulase | Y | Met; CK; Cys |
| endo-1,3(4)-beta-glucanase | XP_748630.1 | 0.2035 | 3 | 56 | 31087.4 | GH16 | Cellulase | Y | Met; CK; Cys |
| GPI-anchored cell wall beta-1,3-endoglucanase EglC | XP_748349.1 | 0.2735 | 11 | 185 | 44650.7 | GH1 | Cellulase | Y | Met; CK; Cys |
| endoglucanase | XP_751043.1 | 0.6499 | 40 | 2577 | 42646.2 | GH5 | Cellulase | Y | Met; CK; Cys |
| endoglucanase | XP_748895.1 | 0.7308 | 41 | 1602 | 25785.3 | GH12 | Cellulase | Y | Met; CK; Cys |
| endoglucanase | XP_747897.1 | 0.7065 | 53 | 782 | 48205.1 | GH7 | Cellulase | Y | Met;CK;Cys |
| beta-glucosidase | XP_753108.1 | 0.3797 | 21 | 326 | 82680.6 | GH3 | Cellulase | Y | Met; CK; Cys |
| 1,4-beta-D-glucan-cellobiohydrolyase | XP_751044.1 | 0.99 | 215 | 10922 | 56456.9 | GH7 | Cellulase | Y | Met; CK; Cys |
| endoglucanase | XP_747057.1 | 0.3308 | 15 | 223 | 85234.3 | GH5 | Cellulase | Y | Met; CK; Cys |
| beta-D-glucoside glucohydrolase | XP_748896.1 | 0.3532 | 15 | 122 | 78380.6 | GH3C | Cellulase | Y | Met; CK; Cys |
| beta-glucosidase | XP_753926.1 | 0.2823 | 18 | 104 | 87391.9 | GH3 | Cellulase | Y | Met; CK; Cys |
| cellobiohydrolase | XP_748511.1 | 0.7048 | 30 | 1020 | 47795.1 | GH6 | Cellulase | Y | Met; CK; Cys |
| endo-1,4-beta-glucanase | XP_748707.1 | 0.3129 | 12 | 133 | 35527.1 | GH61 | Cellulase | Y | Met; CK; Cys |
| alpha-galactosidase | XP_001481538.1 | 0.359 | 17 | 124 | 71594.5 | GH27 | Cellulase | Y | Met; CK; Cys |
| cellobiohydrolase celD | XP_750600.1 | 0.9823 | 91 | 4920 | 48134.7 | GH7 | Cellulase | Y | Met; CK; Cys |
| alpha,alpha-trehalose glucohydrolase TreA/Ath1 | XP_748551.1 | 0.1586 | 11 | 95 | 117017 | GH65N | Cellulase | Y | Met; CK; Cys |
| endoglucanase | XP_752040.1 | 0.86 | 38 | 1055 | 26142 | GH61 | Cellulase | Y | Met; CK; Cys |
| beta-glucosidase | XP_750327.1 | 0.2806 | 17 | 107 | 94752 | GH3 | Cellulase | Y | Met; CK; Cys |
| alpha-glucuronidase | XP_753219.1 | 0.0381 | 2 | 2 | 93658.4 | GH67 | Hemicellulase | Y | Cys |
| beta-xylosidase XylA | XP_753060.1 | 0.1843 | 10 | 29 | 86385 | GH3 | Hemicellulase | Y | Met; CK |
| endo-1,4-beta-xylanase (XynG1) | XP_751100.1 | 0.1222 | 3 | 68 | 23809.7 | GH11 | Hemicellulase | Y | Met; CK |
| extracellular endo-1,4-beta-xylanase | XP_751877.1 | 0.7407 | 35 | 1056 | 35449.8 | GH10 | Hemicellulase | Y | Met; CK; Cys |
| endo-1,4-beta-xylanase | XP_748367.1 | 0.4569 | 34 | 407 | 33080.4 | GH11 | Hemicellulase | Y | Met; CK; Cys |
| mannosidase MsdS | XP_752825.1 | 0.5152 | 22 | 395 | 53839.8 | GH47 | Hemicellulase | Y | Met; CK; Cys |
| endo-1,4-beta-xylanase | XP_751237.1 | 0.7809 | 62 | 2659 | 42136 | GH10 | Hemicellulase | Y | Met; CK; Cys |
| beta-xylosidase | XP_748529.1 | 0.2153 | 11 | 24 | 83336.9 | GH3C | Hemicellulase | Y | Met; CK; Cys |
| endo-1,4-beta-xylanase (XlnA) | XP_748354.1 | 0.6754 | 30 | 3093 | 24493.6 | GH11 | Hemicellulase | Y | Met; CK; Cys |
| endo-1,4-beta-xylanase | XP_754103.1 | 0.3398 | 8 | 42 | 39895.6 | GH10 | Hemicellulase | Y | Met; CK; Cys |
| extracellular endo-polygalacturonase | XP_747009.1 | 0.0495 | 2 | 2 | 37690.9 | GH28 | Pectinase | Y | CK |
| alpha-glucosidase AgdA | XP_751811.1 | 0.0931 | 5 | 8 | 108506.4 | GH31 | Cellulase | Y | CK; Cys |
| alpha- amylase | XP_748411.1 | 0.3048 | 10 | 43 | 62294 | CBM20 | amylase | Y | CK; Cys |
| alpha-glucosidase | XP_752991.1 | 0.0204 | 1 | 2 | 98887.4 | GH31 | Cellulase | Y | Cys |
| alpha-L-arabinofuranosidase A | XP_755850.1 | 0.2027 | 8 | 25 | 71682.9 | GHb | Hemicellulase | Y | Cys |
| alpha-1,2-mannosidase | XP_749038.1 | 0.0442 | 2 | 6 | 87659.1 | GH92 | Hemicellulase | Y | Met |
| beta-1,6-glucanase Neg1 | XP_747510.1 | 0.3156 | 9 | 97 | 51429.9 | GH30 | Cellulase | Y | Met; CK |
| alpha-1,3-glucanase/mutanase | XP_749530.1 | 0.4657 | 23 | 298 | 54022.2 | GH71 | Cellulase | Y | Met; CK; Cys |
| glucan 1,4-alpha-glucosidase | XP_749206.1 | 0.3265 | 17 | 186 | 67099.6 | GH15 | Cellulase | Y | Met; CK; Cys |
| alpha-amylase | XP_749208.1 | 0.1587 | 8 | 81 | 68495.4 | CBM20 | amylase | Y | Met; CK; Cys |
| extracellular polygalacturonase | XP_753090.1 | 0.4497 | 14 | 79 | 38424.5 | GH28 | Pectinase | Y | Met; CK; Cys |
| extracellular exo-polygalacturonase | XP_747778.1 | 0.2172 | 7 | 69 | 48489 | GH28 | Pectinase | Y | Met; CK; Cys |
| N,O-diacetyl muramidase | XP_750902.1 | 0.4518 | 8 | 193 | 24639.1 | GH25 | Hemicellulase | Y | Met; CK; Cys |
| alpha-L-arabinofuranosidase | XP_755608.1 | 0.5602 | 12 | 97 | 36428.5 | GH62 | Hemicellulase | Y | Met; CK; Cys |
| arabinogalactan endo-1,4-beta-galactosidase GalA | XP_750440.1 | 0.1522 | 4 | 31 | 41940.4 | GH53 | Hemicellulase | Y | Met; CK; Cys |
| glycosyl hydrolase | XP_751454.1 | 0.3051 | 9 | 67 | 36755.1 | GH3 | Cellulase | Y | Met; CK; Cys |
| class III chitinase | XP_747991.1 | 0.0352 | 1 | 1 | 40307 | GH18 | Chitinase | Y | CK |
| class V chitinase Chi100 | XP_747948.2 | 0.0197 | 1 | 3 | 150846.1 | GH18 | Chitinase | Y | CK |
| class V chitinase | XP_754491.2 | 0.1333 | 4 | 14 | 49095.6 | GH18 | Chitinase | Y | CK; Cys |
| class V chitinase | XP_001481609.1 | 0.6455 | 25 | 410 | 46493.9 | GH18 | Chitinase | Y | CK; Cys |
| class V chitinase | XP_751248.1 | 0.0247 | 2 | 4 | 146161.1 | GH18 | Chitinase | Y | Met; CK |
| chitosanase | XP_747113.1 | 0.1074 | 2 | 12 | 25330.8 | GH75 | Chitosanase | Y | Met; CK |
| endo-chitosanase, pseudogene | XP_746329.1 | 0.5042 | 12 | 182 | 25191 | GH75 | Chitosanase | Y | Met; CK |
| class III chitinase ChiA1 | XP_747968.1 | 0.0495 | 3 | 16 | 88627.4 | GH18 | Chitinase | Y | Met; CK; Cys |
| class V chitinase ChiB1 | XP_747065.1 | 0.6513 | 33 | 995 | 47621.7 | GH18 | Chitinase | Y | Met; CK; Cys |
| extracellular chitosanase CsnC | XP_754126.2 | 0.2384 | 5 | 33 | 35139.7 | GH75 | Chitosanase | Y | Met; CK; Cys |
| triacylglycerol lipase (LipA) | XP_746588.1 | 0.0417 | 2 | 3 | 66320.4 | CE family | Esterase | Y | CK |
| phytase | XP_746808.1 | 0.1917 | 7 | 25 | 57047.8 | histidine acid phosphatase family | Esterase | Y | CK |
| extracellular GDSL-like lipase/acylhydrolase | XP_749219.1 | 0.1882 | 6 | 14 | 47701.1 | XynE like | Esterase | Y | CK;Cys |
| esterase | XP_001481678.1 | 0.2316 | 4 | 20 | 28770.2 | faeC family. | Esterase | Y | CK; Cys |
| cutinase | XP_755273.1 | 0.1611 | 2 | 8 | 21856 | cutinase family | Esterase | Y | CK; Cys |
| acid phosphatase | XP_746350.1 | 0.1058 | 2 | 5 | 30458.8 | SurE | Esterase | Y | CK; Cys |
| rhamnogalacturonan acetylesterase RgaE | XP_754175.1 | 0.6347 | 11 | 135 | 28920.3 | SGNH hydro | Esterase | Y | CK; Cys |
| extracellular lipase | XP_746953.1 | 0.21 | 8 | 32 | 60745.3 | CE family | Esterase | Y | Cys |
| lipase | XP_749106.1 | 0.0259 | 1 | 1 | 49244.4 | lipase | Esterase | Y | Cys |
| feruloyl esterase | XP_731510.1 | 0.2292 | 6 | 10 | 57675.1 | tannase family | Esterase | Y | Cys |
| cholinesterase | XP_746761.1 | 0.0641 | 4 | 7 | 79233.7 | CE family | Esterase | Y | Cys |
| acid sphingomyelinase | XP_752162.1 | 0.0463 | 2 | 2 | 68419.4 | acid sphingomyelinase family | Esterase | Y | Cys |
| alpha/beta fold family hydrolase | XP_752505.1 | 0.1575 | 3 | 8 | 31847.8 | AB hydrolase-1 | Esterase | Y | Cys |
| lactonohydrolase | XP_752266.1 | 0.2871 | 8 | 20 | 44561.8 | YvrE | Esterase | Y | Cys |
| Ser/Thr protein phosphatase family | XP_748733.1 | 0.0719 | 3 | 4 | 71304.6 | Metallopho | Esterase | Y | Cys |
| GDSL lipase/acylhydrolase family protein | XP_749200.1 | 0.1254 | 3 | 5 | 39576 | Lipase GDSL | Esterase | Y | Cys |
| acid phosphatase PHOa | XP_750107.1 | 0.1741 | 6 | 15 | 49116.1 | acid phosphatase PHOa | Esterase | Y | Met; CK |
| extracellular lipase | XP_748138.1 | 0.4047 | 11 | 115 | 31442.9 | Lipase 3 | Esterase | Y | Met; CK; Cys |
| cellulose-binding GDSL lipase/acylhydrolase | XP_749187.1 | 0.1195 | 3 | 100 | 41800.3 | CBM1 | Esterase | Y | Met; CK; Cys |
| acid phosphatase | XP_746553.1 | 0.2343 | 8 | 45 | 46131.3 | Phosphoesterase | Esterase | Y | Met; CK; Cys |
| acetyl xylan esterase (Axe1) | XP_748362.1 | 0.2329 | 6 | 28 | 29275.1 | CBM1 | Esterase | Y | Met; CK; Cys |
| acetyl xylan esterase | XP_747458.1 | 0.2588 | 11 | 276 | 39252.2 | CE1 family. | Esterase | Y | Met; CK; Cys |
| feruloyl esterase | XP_750792.1 | 0.1977 | 6 | 45 | 57864.5 | tannase family | Esterase | Y | Met; CK; Cys |
| lysophospholipase Plb1 | XP_751952.1 | 0.1264 | 7 | 30 | 68143 | lysophospholipase family. | Esterase | Y | Met; CK; Cys |
| extracellular guanyl-specific ribonuclease RntA | XP_746509.1 | 0.5191 | 4 | 50 | 14032.4 | fungal RNase | Esterase | Y | Met; CK; Cys |
| lysophospholipase A | XP_755409.1 | 0.0936 | 3 | 24 | 38499.1 | fatty acyltransferase like | Esterase | Y | Met; CK; Cys |
| conidial pigment biosynthesis oxidase Abr1/brown 1 | XP_756089.2 | 0.2184 | 10 | 23 | 73445 | multicopper oxidase family | Oxidoreductase | Y | CK; Cys |
| tyrosinase | XP_748428.1 | 0.1697 | 5 | 7 | 42033.7 | Chitin-binding type-4 | Oxidoreductase | Y | Cys |
| isoamyl alcohol oxidase | XP_746836.1 | 0.0346 | 4 | 8 | 111763.1 | oxygen-dependent FAD-linked oxidoreductase | Oxidoreductase | Y | Cys |
| oxalate decarboxylase | XP_755793.1 | 0.1589 | 5 | 33 | 49476.7 | oxalate decarboxylase family | Oxidoreductase | Y | Met; CK |
| FAD-dependent oxidase | XP_749848.1 | 0.0948 | 4 | 10 | 53642.5 | oxygen-dependent FAD-linked oxidoreductase | Oxidoreductase | Y | Met; CK |
| extracellular dioxygenase | XP_754163.1 | 0.1063 | 2 | 7 | 38898.4 |  | Oxidoreductase | Y | Met; CK |
| isoamyl alcohol oxidase | XP_001481692.1 | 0.3552 | 16 | 173 | 60519.5 | oxygen dependent fad linked oxidoreductase | Oxidoreductase | Y | Met; CK; Cys |
| thioredoxin reductase | XP_747990.1 | 0.5 | 21 | 163 | 42850 | TrxB | Oxidoreductase | Y | Met; CK; Cys |
| oxidoreductase, FAD-binding | XP_755781.1 | 0.3277 | 9 | 31 | 50493.8 | FAD-binding PCMH-type | Oxidoreductase | Y | Met; CK; Cys |
| FAD-dependent oxygenase | XP_748405.1 | 0.2347 | 8 | 51 | 55025.8 | FAD-binding PCMH-type | Oxidoreductase | Y | Met; CK; Cys |
| conidial pigment biosynthesis oxidase Arb2 | XP_756088.2 | 0.1533 | 6 | 69 | 65288.9 | multicopper oxidase family. | Oxidoreductase | Y | Met; CK; Cys |
| FAD/FMN-containing isoamyl alcohol oxidase MreA | XP_747715.1 | 0.3292 | 11 | 79 | 61365.2 | oxygen-dependent FAD-linked oxidoreductase | Oxidoreductase | Y | Met; CK; Cys |
| pectate lyase A | XP_749213.1 | 0.4393 | 14 | 169 | 33821.2 | PL1 family. | Pectinase | Y | CK; Cys |
| endo-arabinase | XP_755807.1 | 0.3519 | 8 | 52 | 35614.7 | GH43 | Pectinase | Y | CK; Cys |
| pectate lyase | XP_754136.1 | 0.1504 | 4 | 7 | 42595.5 | PL1 family. | Pectinase | Y | Cys |
| pectate lyase | XP_749862.1 | 0.3632 | 4 | 26 | 24743.5 | PL1 family. | Pectinase | Y | Cys |
| rhamnogalacturonase | XP_747123.1 | 0.0465 | 2 | 2 | 62235.9 | CBM-like | Pectinase | Y | Cys |
| pectin lyase | XP_753604.1 | 0.1211 | 3 | 8 | 39783.1 | PL1 family. | Pectinase | Y | Met; CK |
| rhamnogalacturonase B | XP_746565.1 | 0.286 | 8 | 31 | 56735.6 | PL4 family | Pectinase | Y | Met; CK; Cys |
| pectin methylesterase | XP_747487.1 | 0.2438 | 6 | 78 | 34557.3 | pectinesterase family | Pectinase | Y | Met; CK; Cys |
| 1,3-beta-glucanosyltransferase Gel2 | XP_751022.1 | 0.1137 | 4 | 10 | 51717.2 | GH72 | Transferase | Y | CK; Cys |
| glycosyl hydrolase | XP_748356.1 | 0.0636 | 2 | 6 | 50245.3 | GH76 | Transferase | Y | Cys |
| 1,3-beta-glucanosyltransferase Gel3 | XP_755615.1 | 0.2757 | 13 | 87 | 57078.9 | GH72 | Transferase | Y | Met; CK |
| 1,3-beta-glucanosyltransferase Bgt1 | XP_752511.1 | 0.4033 | 15 | 79 | 33083.8 | GH17 | Transferase | Y | Met; CK |
| 1,3-beta-glucanosyltransferase | XP_746993.1 | 0.0819 | 4 | 16 | 57489.9 | GH72 | Transferase | Y | Met; CK |
| 1,3-beta-glucanosyltransferase Gel1 | XP_749253.1 | 0.5133 | 19 | 89 | 48076.9 | GH72 | Transferase | Y | Met; CK; Cys |
| 1,3-beta-glucanosyltransferase | XP_749664.1 | 0.3522 | 19 | 163 | 58872 | GH72 | Transferase | Y | Met; CK; Cys |
| glycosyl hydrolase family 43 protein | XP_746955.1 | 0.222 | 6 | 62 | 49666.2 | GH43 | Hemicellulase | Y | Met; CK; Cys |
| cellulase family protein | XP_753201.1 | 0.602 | 16 | 126 | 43233.8 | GH5 | Cellulase | Y | Met; CK; Cys |

1. * To whom correspondence should be addressed: [liudongyang@njau.edu.cn](mailto:liudongyang@njau.edu.cn); Tel: +86 25 84396853; Fax: +86 25 84395212, College of Resources and Environmental Science, Nanjing Agricultural University, Nanjing, CHINA.

   [↑](#footnote-ref-1)
